# Chinese Herbal Medicine for Mild Cognitive Impairment: A Systematic Review of Randomized Controlled Trials

**DOI:** 10.3389/fneur.2022.903224

**Published:** 2022-06-30

**Authors:** Ning Liang, Yaxin Chen, Sihong Yang, Changhao Liang, Lidong Gao, Shang Wang, Yanping Wang, Zhanjun Zhang, Nannan Shi

**Affiliations:** ^1^Institute of Basic Research in Clinical Medicine, China Academy of Chinese Medical Sciences, Beijing, China; ^2^Center for Evidence-Based Chinese Medicine, Beijing University of Chinese Medicine, Beijing, China; ^3^College of Traditional Chinese Medicine, Tianjin University of Traditional Chinese Medicine, Tianjin, China; ^4^State Key Laboratory of Cognitive Neuroscience and Learning, Beijing Normal University, Beijing, China

**Keywords:** mild cognitive impairment (MCI), Chinese herbal medicine, systematic review, meta-analysis, traditional Chinese medicine

## Abstract

**Objectives:**

This study aims to explore the benefits and harms of Chinese Herbal Medicine (CHM) for mild cognitive impairment (MCI).

**Methods:**

Electronic searching was conducted in two English and four Chinese databases till 2021 December. Randomized clinical trials on CHM compared to no intervention, placebo or other therapies for MCI were included.

**Results:**

Forty-nine RCTs (48 finished trials and 1 protocol) were identified. The overall methodological quality of included trials was relatively low. This review found that compared to no intervention or placebo, CHM can significantly decrease the number of patients who progressed to dementia (RR 0.36, 95% CI 0.22–0.58) and increase the cognitive function assessed by MMSE (MD 1.96, 95% CI 1.41–2.50) and MoCA (MD 2.44, 95% CI 1.57–3.31). The subgroup analysis of different CHM showed that Ginko leaf tablets can significantly improve the cognitive function compared to no intervention or placebo when assessed by MMSE (MD 2.03, 95% CI 1.18–2.88) and MoCA (MD 3.11, 95% CI 1.90–4.33). Compared to western medicine, CHM can significantly increase the score of MMSE (MD 0.88 95% CI 0.46–1.30) and MoCA (MD 0.87, 95% CI 0.33–1.41), but there was no significant difference on the score of ADL (SMD −0.61, 95% CI −1.49 to 0.27). None of the RCTs reported on the quality of life. Of 22 RCTs that reported adverse events, there was no statistical difference between the CHM and the control group.

**Conclusions:**

CHM, Ginko leaf extracts in particular, could help to prevent progression into dementia and to improve cognitive function and ability of daily living activities. More qualified RCTs were needed to confirm the conclusion due to the low quality of current trials.

**Systematic Review Registration:**

Unique Identifier: CRD42020157148.

## Introduction

Mild cognitive impairment (MCI) represents a transitional state between normal aging and dementia (1). It is diagnosed by the presence of one or more domains of cognitive impairment without fulfilling the diagnostic criteria for dementia (2). If impairment involves memory, it is known as amnestic MCI, and if not, non-amnestic MCI (2). The population-based studies showed that the frequency of MCI is estimated to be 15–20% in persons 60 years and older (3). The annual rate of development of dementia in people with MCI varied between 5 and 15% (3). People with MCI are at higher risk of progression to dementia than age-matched general people, therefore, it is critical to find treatments that may improve symptoms or prevent or delay progression to dementia (2).

Currently, no treatments, pharmacologic or non-pharmacologic, are approved specifically for MCI by the Food and Drug Administration (2). Doctors sometimes prescribe approved drugs that were for Alzheimer's disease to patients with MCI, such as cholinesterase inhibitors, but according to the practice guideline by the American Academy of Neurology, doctors may not choose cholinesterase inhibitors and must first discuss lack of evidence when offering it to people with MCI (2). The guideline found evidence on 12 pharmacologic treatments (e.g., Donepezil, Galantamine, Rivastigmine), but none was recommended to be used for MCI as no high-quality evidence exists (2). For non-pharmacologic treatments, exercise training and cognitive training may help improve cognitive measures (2).

In recent years, Chinese herbal extracts and preparations have received considerable research attention for the management of cognition impairment. Previously published systematic reviews of Chinese herbal medicine (CHM) for MCI have been limited by enrolling people with mixed cognitive impairment (including MCI and other memory impairment), or focusing only on Montreal Cognitive Assessment (MoCA) (4), Mini-mental state examination (MMSE) (5), or Alzheimer's Disease Assessment Scale-Cognitive subscale (ADAS-Cog) (5), but not on suggested clinically meaningful patient outcomes such as development into dementia or quality of life (5). These reviews were likely not to provide dependable results because of possible flaws in methodology, such as lack of protocol (4–6), the inadequate technique for risk of bias assessment (6), or synthesis of data regardless of significant clinical heterogeneity (5). An update of evidence is also warranted as these systematic reviews were based on evidence up till December 2017 (4–6). Therefore, this study was conducted to summarize the up-to-date evidence and to explore the benefits and harms of CHM for MCI in a systematic review of randomized clinical trials (RCTs) with rigorous and reasonable methodology.

## Methods

The protocol of this review was registered on PROSPERO (registration number: CRD42020157148). We followed the guidelines provided in the Cochrane Handbook for Systematic Reviews of Interventions (7).

### Inclusion Criteria

#### Types of Studies

We searched for RCTs regardless of language, publication year, publication format, and publication status.

#### Types of Participants

We included participants of any gender and at any age diagnosed as MCI by trialists or according to guidelines.

#### Types of Interventions

We included CHM at any dose and form, compared with placebo, no intervention or other commonly used therapies (e.g., western medication, cognitive training, or lifestyle change) for MCI. CHM with fixed production process and controllable quality, approved by national medical products administration and state administration for market regulation (SAMR) were included. Comparison between two different CHM was excluded. We allowed co-intervention when it was administered equally to the intervention and the control group.

#### Types of Outcome Measures

Primary outcomes were the number of participants progressing into dementia, cognitive function assessed by authority scales such as MoCA and MMSE, and the number of participants with adverse events. Secondary outcomes were quality of life and assessment of behavior or psychiatric symptoms.

### Search Strategy

We searched MEDLINE, Cochrane CENTRAL, China National Knowledge Infrastructure (CNKI), Chongqing VIP (CQVIP), Wanfang Data, and Sinomed from their inception to 31 December 2021. We also searched the reference lists of the meta-analyses on this topic and the references of the included trials. The search strategy (see Supplementary Table 1 for details) included the following key medical keywords: “mild cognitive impairment”, “Chinese herbal medicine”, “Chinese patent medicine”, “random”, “randomization”, and “randomized clinical trials”.

### Data Selection and Extraction

Reviewers in pairs (SH Yang, CH Liang, LD Gao, S Wang) independently screened titles and abstracts to retrieve the potentially eligible trials, and read full-text papers to identify the trials that should be included. Reviewers (YX Chen, SH Yang) extracted the information from the included trials through a pre-piloted table created in Microsoft Excel. Another author (N Liang) rechecked the extracted information. The extracted information included publication data (e.g., publication year, authors); study characteristics and design; characteristics of the participants (e.g., age, gender, diagnostic and inclusion criteria); interventions and controls (e.g., dose, form, duration); and outcomes. We extracted data at maximum follow-up.

### Assessment of Risk of Bias in Included Trials

Reviewers in pair (SH Yang, CH Liang, LD Gao, S Wang) independently assessed the risk of bias according to the Cochrane “risk of bias” tool (7). We evaluated the following items: allocation sequence generation, allocation concealment, blinding of participants and personnel, blinding of outcome assessment, incomplete outcome data, selective outcome reporting, and other bias.

### Statistical Analysis

RevMan 5.3 was used to perform statistical analyses. We used the risk ratio (RR) for measuring dichotomous outcomes and mean difference (MD) for continuous data, with 95% confidence intervals (CIs). When studies used different instruments to measure the same continuous outcome, we calculated the standard mean difference (SMD), with 95% CI. We assessed clinical and methodological heterogeneity by carefully examining trial participant and intervention characteristics and the design of the included trials. We assessed our intervention effects with both fixed-effect model and random-effects model, and we reported both results when results differed (e.g., one giving a significant intervention effect, while the other no significant intervention effect); otherwise, we reported the estimate closest to the zero effect (the highest *P*-value). We started by looking at the forest plots for signs of statistical heterogeneity. Next, we used the Chi^2^ test with a significance threshold set as *P* < 0.10 and measured the amount of heterogeneity with the I^2^ statistic to assess to what extent variation is from heterogeneity rather than chance. We interpreted the I^2^ statistic as suggested in Cochrane Handbook: 0–40%: might not be important; 30–60%: might represent moderate heterogeneity; 50–90%: might represent substantial heterogeneity; 75–100%: considerable heterogeneity (7). In case of available data, we performed the subgroup analyses in terms of different interventions and controls, and different causes of MCI. We used sensitivity analyses whenever we wanted to test the robustness of our findings. We planned to assess reporting bias using funnel plots if we obtained data from at least 10 trials per comparison. To assess the risk of publication bias, we intended to look for symmetry or asymmetry of each funnel plot.

## Results

### Description of the Search

**Figure 1:**
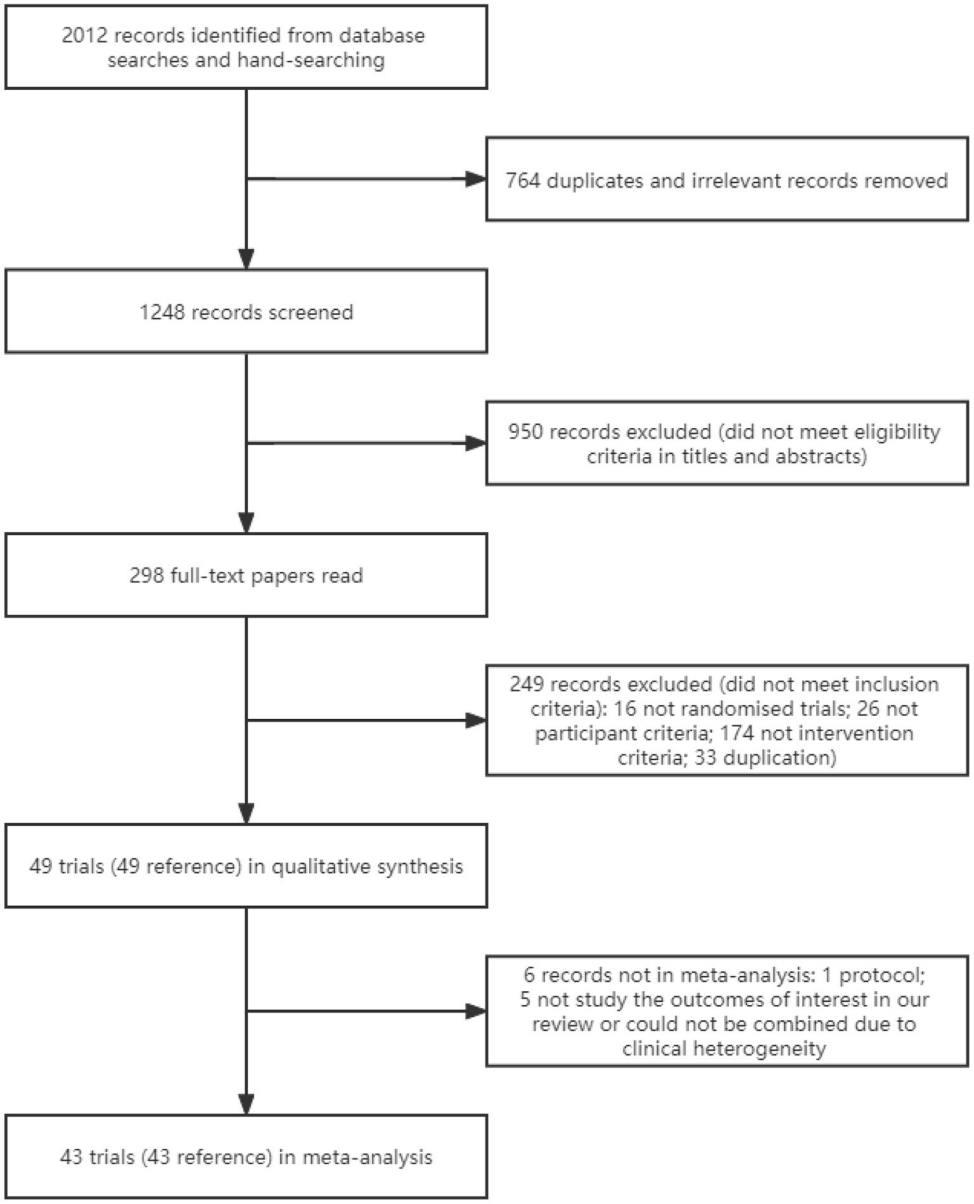
Flow chart of included studies in this systematic review.

We identified 2,012 references through searching the databases and the reference lists of meta-analyses on this topic and included trials (Figure 1). After excluding duplicates and irrelevant studies, titles and abstracts of 1,248 references were screened; of these, 298 full-text papers were read. Totally, 49 references of 49 trials were included (8–56).

### Basic Characteristics of Included Studies

We included 48 finished trials and one protocol (30). Of the 48 finished trials, 43 trials provided data for meta-analyses. The remaining five trials either did not study the outcomes of interest in our review or could not be combined due to clinical heterogeneity (19, 34, 38, 53, 55); hence we used the provided information only in a narrative way. Fifteen trials received funding from government or academic institution; four trials were funded by pharmaceutical companies, and high risk of financial supports was suspected even though no conflict of interest was declared (33, 42, 48, 53); the remaining 30 trials did not provide information on funding. Undisclosed funding may influence trial results and may lead to poor trial design.

Sample size ranged from 18 to 245 participants, and a total of 4,656 participants were randomized. For 42 trials that reported the gender of participants, the total ratio of female to male was 1,897:1,892. Age of participants was reported in 44 trials, and mean age of participants in these trials was 67.82 years old (Table 1).

**Table 1:** Characteristics of trials included in the review.

**Study ID**	**Participants**	**CHM group**	**Control group**	**Sample size (I/C), dropout (I/C)**	**Age (I/C)**	**No. of female (I/C)**	**Outcome measures**
Su et al. (15)	MCI	Ginkgo leaf tablets, 19.2 mg, t.i.d; + Donepezil hydrochloride; 2 m	Donepezil hydrochloride, 5 mg (1st month); then 10 mg; 2 m	100 (50/50), 0	53.5 ± 8.5/51.8 ± 9.6	44/100	MoCA; EEG; ERP P300
Dong (19)	MCI	Ginkgo leaf tablets, 19.2 mg, t.i.d; + Donepezil hydrochloride; 2 m	Donepezil hydrochloride, 5 mg, q.d, 2 m	114 (57/57), 0	66.2 ± 2.6/65.4 ± 2.5	48/114	MMSE; BI; SAS; SDS; symptom score (fail to recognize; visual space disorder; misuse; aphasia; memory)
Xiao (23)	MCI	Ginkgo leaf tablets, 19.2 mg, t.i.d; + Basic treatment; 12 m	Basic treatment (health education, moderate exercise, nutrition support), 12 m	113 (58/55), 0	72.78 ± 5.03/73.53 ± 5.97	73/113	MMSE; ADL; CDT; WMS; number of patients progressed to dementia
Han (14)	MCI	Ginkgo leaf tablets, 1 tablet, t.i.d; +Basic treatment; 12 m	Basic treatment (health education, moderate exercise, nutrition support), 12 m	120 (60/60), 0	72.3 ± 5.3/71.7 ± 4.9	79/120	MQ; number of patients progressed to dementia; MMSE; serum index (MDA, AchE); AE
Zhang (27)	Vascular MCI	Compound Congrong Yizhi, 4 capsules, t.i.d, 6 m	No intervention	18 (9/9), 0	60–69: 6/4 70–80: 3/5	7/18	MoCA; TCM symptom score; AE
Wang (20)	Post-stroke MCI	Compound Congrong Yizhi, 4 capsules, t.i.d; +Health education; 6 m	Health education	20 (10/10), 0	64.1 ± 5.9	5/20	MMSE; MoCA; ADL
Ye (21)	Amnestic MCI	Tongxinluo, 6 capsules, add to 12 capsules if no AE; + Memantine; 6 m	Memantine, 5 mg, add to 20 mg if no AE, 6 m	70 (35/35), 7 (5/2)	69.8 ± 6.3/68.5 ± 5.7	37/70	MMSE; DSR; AE
Jia et al. (25)	MCI	Tongxinluo, 2-4 capsules, b.i.d; + Oxiracetam; 1 m	Oxiracetam, 800 mg, b.i.d, 1 m	48 (25/23), 0	72 ± 4/70 ± 5	21/48	Cognitive function; CGI-EI; number of patients progressed to dementia; AE
Huang et al. (17)	MCI	Liuwei Dihuang, 8 pills, t.i.d; +Cognitive training; 12 m	Cognitive training, 12 m (2 m treatment then 1 m rest)	86 (43/43), 48 (24/24)	79.5 ± 7.80	62/86	MoCA; AE
Liu et al. (30)	Vascular MCI	Yangxue Qingnao granules, 4 g, t.i.d; +Basic treatment; 4 m	Basic treatment for vascular diseases, 4 m	45 (24/21), 0	76.5/73.5	24/45	MMSE; MQ; AE
Dong (11)	MCI	Gui Ling Ji capsule, 0.6 g, q.d; +Cognitive training; 3 m; follow up of 12 m	Cognitive training, 3 m; follow up of 12 m	100 (50/50), 0	NR	NR	MMSE; ADL
Lin (29)	Vascular MCI	Astragalus injection, 10 mL, q.d; Nimodipine; 1 m	Nimodipine, 30 mg, t.i.d, 1 m	59 (30/29), 0	65.03 ± 6.51/64.27 ± 6.63	28/59	MMSE; CDT; ERP P300; AE; TCM symptom score; serum index (TC, TG, HDL-C, LDL-C, CRP); insuline resistance index
Lan et al. (16)	Vascular MCI	Qizhi Tongluo capsule, 800 mg, b.i.d; Nimodipine; 3 m	Nimodipine, 30 mg, t.i.d, 3 m	60 (30/30), 0	66.22 ± 2.85/67.43 ± 1.86	NR	MMSE; TCM symptom score
Wu et al. (10)	MCI	Tianmeng oral liquid, 20 mL, b.i.d; Citicoline sodium tablets; 2 m	Citicoline sodium tablets, 0.2 g, t.i.d, 2 m	102 (53/49), 0	69.6 ± 7.8	41/102	MMSE; AE; transcranial doppler test; TCM symptoms score
Yu et al. (9)	Amnestic MCI	Compound sea snake capsule, 0.3 g (1st week), 0.6 g (2nd week), then 0.9 g, t.i.d, 24 m	No intervention, 24 m	120 (60/60), 27 (12/15)	76.1 ± 5.9/75.7 ± 5.9	52/93	MMSE; MoCA; ADAS-Cog
Meng (22)	Post-stroke MCI	Compound Congrong Yizhi, 4 capsules, t.i.d, 6 m	Nimodipine, 30 mg, t.i.d, 6 m	60 (30/30), 0	67.19 ± 7.56	29/60	MoCA; ADL- Barthel; AE; core symptom scores
Gao et al. (18)	Post-stroke MCI	Compound Congrong Yizhi, 4 capsules, t.i.d; +Basic treatment; 6 m	Nimodipine, 30 mg, t.i.d; +Basic treatment for stroke; 6 m	140 (70/70), 7 (3/4)	67.1 ± 5.3/66.3 ± 4.9	65/133	MMSE; MoCA; AE
Chen et al. (12)	MCI	Compound Congrong Yizhi, 4 capsules, t.i.d, 3 m	Donepezil hydrochloride, 1 tablet, q.d, 3 m	60 (30/30), 0	67.9 ± 7.6/67.5 ± 8.1	35/60	MMSE; ADAS-Cog; ADL; AE
Ouyang and Ouyang (24)	Ischemic post-stroke MCI	Jiannao Bushen pill, 15 capsules, b.i.d, 2 m	Nimodipine, 60 mg, t.i.d, 2 m	75 (39/36), 0	69.3 ± 7.7/67.6 ± 6.7	27/75	MMSE
Chen et al. (13)	MCI	Shenzhiling oral liquid, one piece, b.i.d, 6 m	Huperzine A tablets, 100 μg, b.i.d, 6 m	69 (35/34), 0	66.46 ± 3.85/65.94 ± 4.10	35/69	MMSE; MoCA; CDT; ADL; ADAS-Cog; RVR; DS; visual reaction time test; hand coordination test
Tian et al. (31)	MCI	Jinsiwei pill, 4 pills, t.i.d; +Piracetam tablets placebo; 3 m	Piracetam tablets, 2 tablets, t.i.d; +Jinsiwei pill placebo; 3 m	60 (30/30), 0	57.23 ± 8.14/60.43 ± 7.05	NR	MMSE; memory function scores
Xie et al. (28)	Amnestic MCI	Tianzhi granules, 5 g, t.i.d, 1 m	Donepezil hydrochloride, 5mg, q.d, 1m	96 (68/28), 5 (5/0)	69 ± 7/65 ± 6	46/96	MMSE; ADL; TCM symptom score
Zhu (26)	MCI	Xuanyunning tablet, 2 tablets, t.i.d, 3 m	Piracetam tablet, 0.8 g, t.i.d, 3 m	100 (50/50), 0	75.38 ± 6.79/76.21 ± 5.98	47/100	MSEE; TCM symptom score; CGI-EI; AE; hippocampal volume
Zhang et al. (8)	Parkinson related MCI	Fufang Huonao Shu, 2 tablets, t.i.d; +Madopar; 6 m	Oxiracetam, 800 mg, t.i.d; + Madopar; 6 m	60 (30/30), 0	65.13 ± 7.85/64.89 ± 8.29	31/60	MMSE; MoCA; ADL; TCM symptom score
Zhang et al. (46)	Parkinson related MCI	Tebonin (Ginkgo biloba extract tablet), 80 mg, t.i.d; +Basic treatment; 6 m	Basic treatment (Madopar +Health education and lifestyle guidance), 6 m	83 (43/40), 4 (1/3)	62.25 ± 8.51	37/83	MMSE; MoCA; number of patients progressed to dementia
Xia (45)	MCI	Tanakan (Ginkgo biloba extract tablet), 40 mg, t.i.d; + Basic treatment;12 m	Basic treatment (Medopar + Nimodipine + health education, exercises and nutrition support), 12 m	245 (123/122), 0	64.1 ± 7.1/63.7 ± 6.5	122/245	MMSE; CDT; RBC-Ach; RBC-AchE; number of patients progressed to dementia; AE
Zhang et al. (36)	MCI	Ginkgo biloba extract injection, 20 mL; + GIK; 14 d	GIK, 250 mL, 14 d	200 (100/100), 0	75.7 ± 7.3/73.1 ± 5.7	NR	MMSE; MoCA; serum index (hs-CRP, IL-6)
Wang et al. (50)	MCI	Styron (Ginkgo biloba extract tablet), 19.2 mg, t.i.d; +Basic treatment; 12 m	Basic treatment (health education and lifestyle guidance), 12 m	87 (45/42), 0	80.86 ± 5.99	NR	MMSE; ERP P300
Zheng and Zheng (34)	MCI	Ginkgo biloba extract tablet, 19.2 mg, t.i.d; +Basic treatment; 12 m	Basic treatment (health education, exercise and nutrition support), 12 m	100 (50/50), 0	66.57 ± 2.56/66.38 ± 2.47	47/100	CDT
Li et al. (44)	MCI with CHD and hyperuricemia	Ginkgo biloba extract tablet, 9.6 mg, t.i.d; +Basic treatment; 3 m	Basic treatment (Allopurinol, 0.1 g, t.i.d; exercise), 3 m	69 (35/34), 0	71.36 ± 4.97/72.58 ± 5.09	39/69	MMSE; CDT; number of patients progressed to dementia
Zhang et al. (40)	SVD related MCI	Ginkgo bilobate dispersible tablets, 0.15 g, t.i.d; +Donepezil +Basic treatment; 6 m	Donepezil, 5 mg, q.d; Basic treatment for primary disease; 6 m	86 (43/43), 0	45–69/46–70	44/86	MMSE; ADL
Zheng et al. (41)	MCI	Yindanxinnaotong soft capsule, 4 capsules, t.i.d; + Aniracetam; 3 m, follow up of 6 m	Aniracetam, 0.2 g, t.i.d, 3 m; follow up of 6 m	149 (75/74), 0	72.7 ± 7.2/71.9 ± 8.5	65/149	MMSE; ADL; TCM symptom score; AE
Cai et al. (37)	MCI with depression	Shuganjieyu capsule, 3 capsules, b.i.d, 6 m	Fluoxetine hydrochloride, 20 mg, 6 m	120 (60/60), 0	66.7 ± 9.6/65.8 ± 10.3	54/120	TCM symptom score; HAMD; MMSE; AE
Zhu and Zhang (53)	MCI	Nao Li Bao pills, 4 pills, t.i.d; + Piracetam tablets placebo	Piracetam tablets, 2 tablets, t.i.d; + Nao Li Bao pills placebo	76 (41/35), 0	59.63 ± 8.23/61.25 ± 8.05	43/76	Effective rate
Yuan et al. (47)	MCI	Huan Shao capsules, 2.1 g, t.i.d, 6 m	Nimodipine, 40 mg, t.i.d, 6 m	76 (38/38), 0	79.85 ± 9.58/78.69 ± 9.32	40/76	MoCA; TCM symptom score; AE
Zhang et al. (38)	Post-stroke MCI	Compound Congrong Yizhi, 4 capsules, t.i.d, 3 m	Nimodipine, 30 mg, t.i.d, 3 m	140 (70/70), 7 (3/4)	67.1 ± 5.3/66.3 ± 4.9	65/133	Vascular endothelial function index; cerebral blood flow index
Zhao and Xiang (51)	Diabetic MCI	Yang Xue Qing Nao granules, 4 g, t.i.d; + Basic treatment; 4 m	Basic treatment, 4 m	70, 0	NR	NR	MMSE; serum index (CRP, LDL-C, SOD)
Gavrilova et al. (42)	Amnestic MCI	Ebb 761 (Ginkgo biloba extracts tablets), 240 mg, q.d, 6 m	Placebo, 240 mg, q.d, 6 m	160 (80/80), 5 (2/3)	65 ± 7/63 ± 7	124/159	STAI-X1; GDS; TMT; CGI; AE; Neuropsychiatric inventory
Steiner et al. (32)	MCI	Sailuotong, 2 capsules, b.i.d, 3 m	Placebo, 2 capsules, b.i.d, 3 m	–	–	–	Logical memory story A; Digit symbol; D-KEFS, RCFS; Neuropsychological test battery; QOL; DASS-21; physiological tests; inflammatory tests
Yakoot et al. (48)	MCI	Memo, 750 mg, q.d, 1 m	Placebo, 750 mg, q.d, 1 m	66 (33/33), 6 (3/3)	65.97 ± 6.52/66.43 ± 5.79	27/60	MMSE; AE
Zhang et al. (35)	Amnestic MCI	Bushen capsule (Congrong Yizhi capsule), 4 capsules, t.i.d, 24 m	Placebo, 4 capsules, t.i.d, 24 m	60 (30/30), 2 (0/2)	64.67 ± 6.83	32/60	MMSE; AVLT; ROCF; Digit Span test; TMT; SDMT; SCWT; CVFT; BNT; AE; MRI
Gschwind et al. (33)	MCI with dual-task-related gait impairment	GBE 1370 (Ginkgo biloba extract), 120 mg, b.i.d, 6 m	Placebo, 120 mg, b.i.d, 6 m	50 (25/25), 8 (24/18)	68.5 ± 8.4	25/50	Spatio-temporal gait parameters; AE
Guo et al. (39)	Vascular MCI	Xinnaoning capsule, 1.35 g, t.i.d; +Basic treatment; 6 m	Nimodipine, 30 mg, t.i.d; +Basic treatment (health education and exercise); 6 m	80 (40/40), 2 (0/2)	70.13 ± 6.02/70.00 ± 5.47	37/78	MMSE; MoCA
Li et al. (49)	MCI	Ginkgo biloba tablets, 80 mg, t.i.d, 3 m	Nicergoline tablets, 10 mg, t.i.d, 3 m	150 (75/75), 0	68.2 ± 4.4	65/150	MMSE
He (43)	Post stroke MCI	Ginkgo Leaf Extract and dipyridamole injection, 20 mL, with sodium chloride; 14 d	Sodium chloride injection, 250 mL, q.d, 14 d	80 (40/40), 0	60.7 ± 5.84/61.35 ± 5.75	30/80	MMSE; MoCA; AE
Li (52)	MCI	Quan Tian Ma capsules, 2.5 g, t.i.d; +Basic treatment; 3 m	Basic treatment, 3 m	60 (30/30), 0	71.6 ± 6.6/71.2 ± 6.7	26/60	MMSE; AE
Guo et al. (54)	MCI with Type 2 Diabetic	Jinlida granules, 9 g, tid; +Sitagliptin, 100 mg, qd; 3 m	Sitagliptin, 100 mg, qd; 3 m	102 (51/51), 6 (3, 3)	67.8 ± 5.4/68.2 ± 5.8	51/102	MMSE; MoCA; symptom score; effective rate; inflammatory tests; AE
Liu (55)	Post stroke MCI	Zhongfeng Huichun tablet, 5 tablets, tid; + Butylphthalide and Sodium, 25 mg, bid; 14 d	Butylphthalide and Sodium Chloride injection, 25 mg, bid; 14 d;	110 (55/55), 0	54.2 ± 2.6/53.8 ± 2.4	49/110	Effective rate (NIHSS); LOTCA
Yang et al. (56)	Vascular MCI	Yinxing Tongzhi tablets, 0.15 g, tid; + Butylphthalide Soft capsules, 0.2 g, tid; 3 m	Butylphthalide Soft capsules, 0.2 g, tid; 3 m	102 (51/51)0.0	57.04 ± 6.81/57.18 ± 6.52	39/102	MMSE; inflammatory tests; ADL; AE

*CHM, Chinese herbal medicine; I, Intervention; C, Control; MCI, Mild cognitive impairment; MMSE, Mini-mental state examination; MoCA, Montreal cognitive assessment; ADL, Activities of daily living scale; IADL, Instrumental activities of daily living scale; ERP P300, Event-related potentials P300; EEG, Electroencephalogram; SAS, Self-rating anxiety scale; SDS, Self-rating depression scale; BI, Barthel index; CDT, Clock drawing task; WMS, Wechsler memory scale; MQ, Memory quotient; MDA, Maloudialdehyde; AchE, Acetylcholinesterase; DSR, Delayed story recall; AE, Adverse events; CGI- EI, Clinical global impression-efficacy index; ADAS-Cog, The Alzheimer's disease assessment scale-cognitive subscale; RVR, Rapid verbal retrieve; DS, Digit span; TCM, Traditional Chinese medicine; TG, Triglyceride; TC, Total cholesterol; HDL-C, High density liptein-cholesterol; LDL-C, Low density lipoprotein-cholesterol; CRP, C-reactive protein; NR, Not reported; NHFPC, National Health and Family Planning Commission; CHD, Coronary heart disease; SVD, Cerebral small vessel disease; HAMD, Hamilton Depression Scale; CACMS, China Academy of Chinese Medical Sciences; IBRCM, Institute of Basic Research in Clinical Medicine; CVFT, Category verbal fluency test; AVLT, Auditory verbal learning test; MRI, Magnetic resonance imaging; STAI-X1, State-Trait anxiety inventory; GDS, Geriatric depression scale; TMT, Trail-Making test; ROCF, Rey-Osterrieth complex figure test; BNT, Boston naming test; SDMT, Symbol digit modalities test; SCWT, Stroop color and word test; RCFS, Rey complex figure test; D-KEFS, Delis-Keplan executive function system; DASS-21, 21-item Depression, Anxiety, Stress scale*.

Twenty-seven CHM were explored in the included trials (see Supplementary Table 2 for details). The dosage form included capsules (*n* = 13), pills (*n* = 4), tablets (*n* = 4), granules (*n* = 3), oral liquids (*n* = 2), and injection (*n* = 2). The duration of CHM treatment ranged from 14 to 24 months.

### Risk of Bias in Included Studies

**Figure 2:**
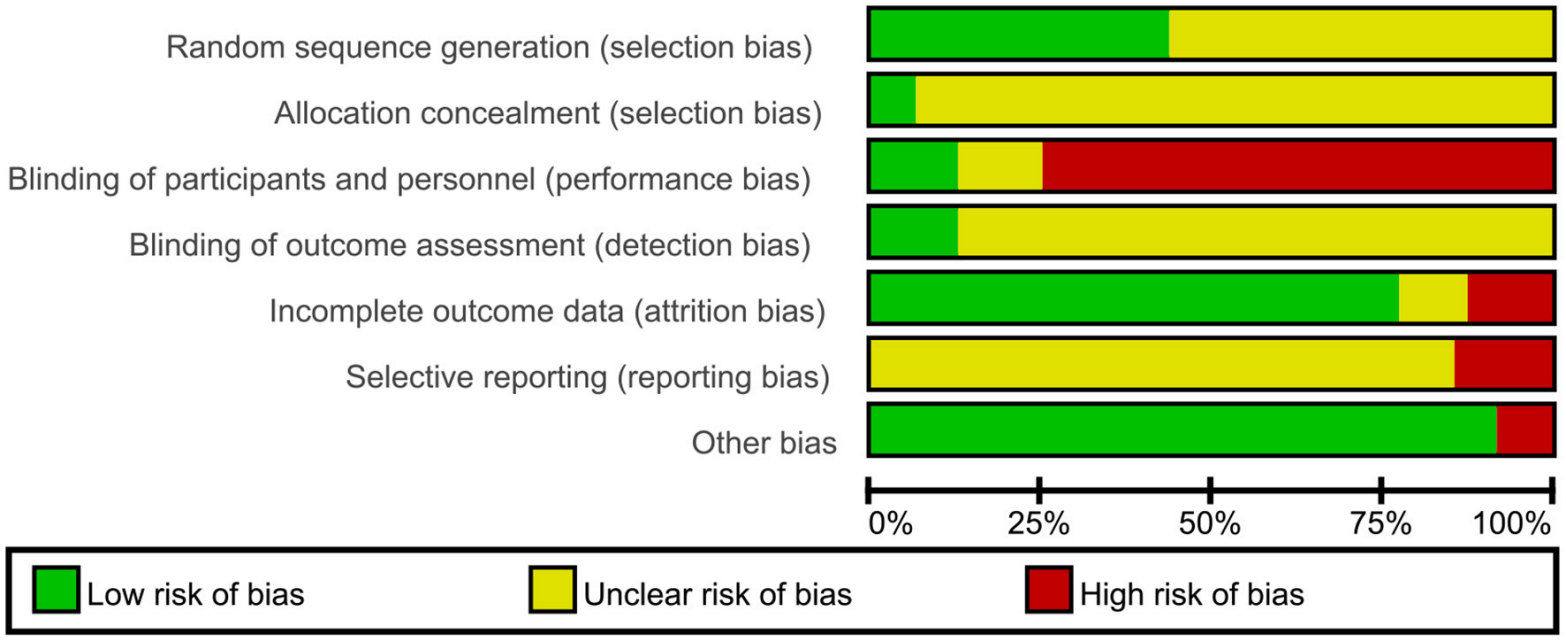
Risk of bias graph: review authors' judgments about each risk of bias item presented as percentages across all included studies.

**Figure 3:**
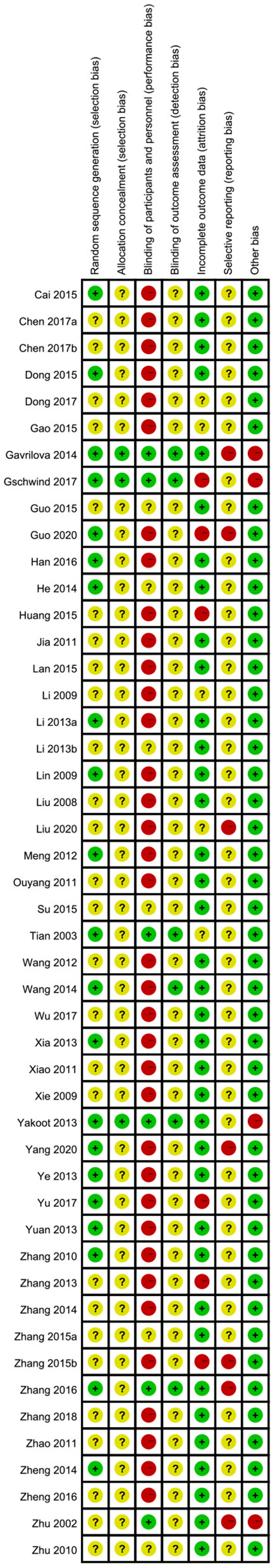
Risk of bias summary: review authors' judgments about each risk of bias item for each included study.

We carried out the risk of bias assessment for 48 finished trials. We judged many trials to have unclear risk of bias in certain domains because we could not obtain additional information from the trial authors after we contacted them (Figures 2, 3).

For selection bias, 21 trials (9, 14, 19, 21, 27, 29, 33, 35, 37, 41–45, 47, 48, 54, 56) which either used SAS software or the randomization table to generate a randomization sequence were assessed at low risk of bias; the remaining 27 trials did not specify the randomization method, and therefore we assessed at unclear risk of bias. The method used to conceal allocation was not reported in most of the included trials, except for three trials (33, 42, 48) assessed at low risk of bias regarding allocation concealment. Six trials (31, 33, 35, 42, 48, 53) adequately performed blinding of participants and personnel; the remaining 42 trials did not adequately describe any blinding methods of participants and personnel; moreover, 36 trials of them were assessed at high risk of bias as CHM in addition to a co-intervention was compared to a co-intervention or the form of CHM was quite different from that of control intervention. Blinding of outcome assessors was adequately performed in six trials (20, 31, 33, 35, 42, 48); the remaining 42 trials did not describe the method used for blinding of outcome assessors. 38 trials reported having no missing data and included all participants in data analyses, showing low risk of attribution bias; six trials (9, 17, 33, 38, 46, 54) had high dropout rates and excluded dropouts from the analyses, so we assessed them at high risk of bias as they did not properly deal with the missing data; four trials (11, 18, 31, 52) did not report the information of dropouts, and therefore were assessed at unclear risk of bias. The risk of selective reporting of one trial was low as the published paper was consistent with the protocol; 40 trials was unclear because of lack of pre-published trial protocols though one primary outcome was reported; seven trials (35, 38, 42, 53–56) was assessed to be of high risk as no pre-published protocols and no primary outcome was reported. Except four trials (33, 42, 48, 53) which was funded by pharmaceutical companies, and resulted our assessments of high risk of bias, the remaining 44 trials appeared to be free of other factors that could put them at risk of bias.

### Effect Estimates

All 48 finished trials employed parallel design, 33 of which compared CHM with no intervention/placebo allowing for co-intervention; the remaining 15 trials (9, 12, 13, 22, 24, 26–28, 33, 35, 37, 38, 42, 47, 48) compared CHM with western drugs allowing for co-intervention.

#### CHM vs. No Intervention/Placebo (Co-intervention Was Allowed)

##### Number of Participants Progressed to Dementia

**Figure 4:**
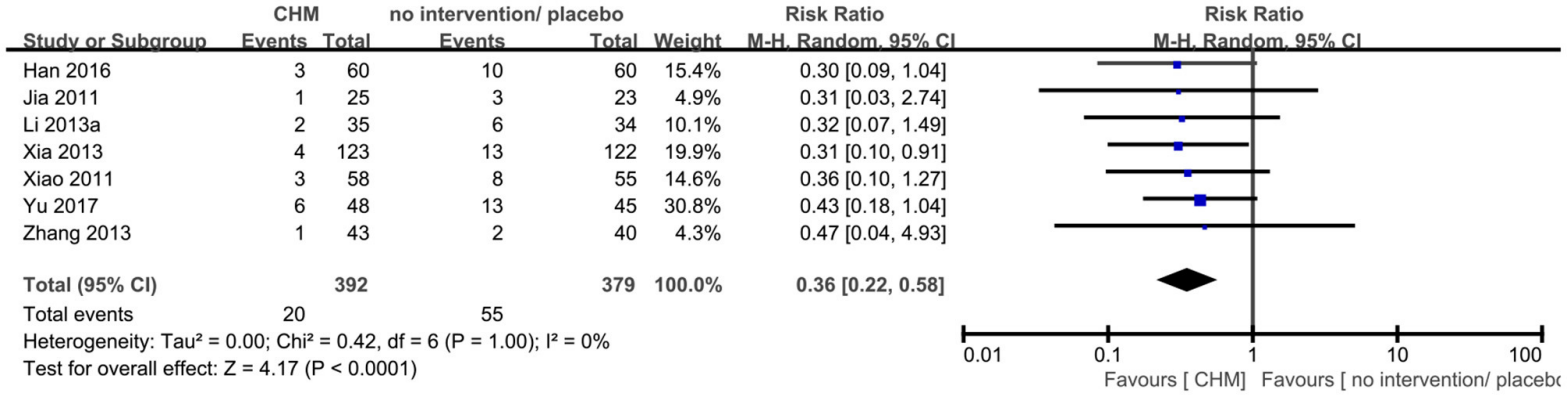
Forest plot of comparison: CHM vs. no intervention/placebo, outcome: number of participants progressed to dementia.

Seven RCTs (9, 14, 23, 25, 44–46) reported this outcome, and the meta-analysis showed that CHM significantly decreased the number of patients who progressed to dementia compared to no intervention or placebo (RR 0.36, 95% CI 0.22–0.58, 7 trials, 771 participants, I^2^ = 0%) (Figure 4).

#### Cognitive Function

**Figure 5:**
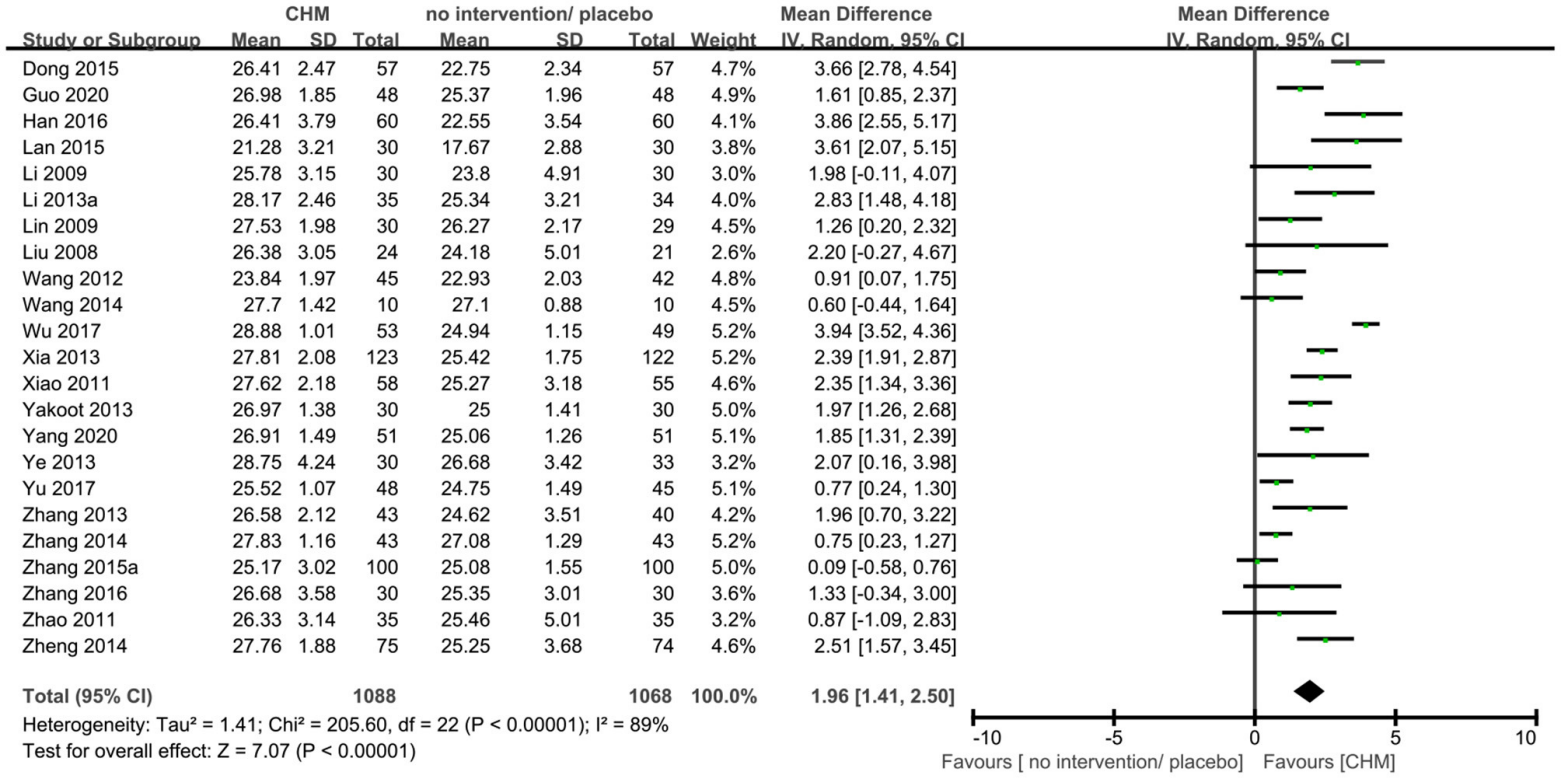
Forest plot of comparison: CHM vs. no intervention/placebo, outcome: MMSE.

**Figure 6:**
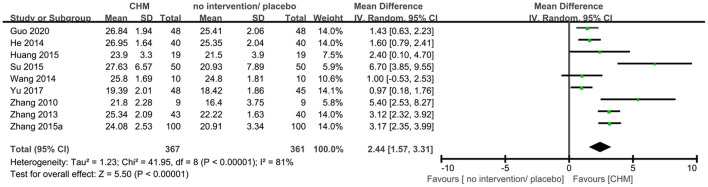
Forest plot of comparison: CHM vs. no intervention/placebo, outcome: MoCA.

Twenty-three trials (9, 10, 14, 16, 19, 20, 23, 25, 29, 30, 35, 36, 40, 41, 44–46, 48, 50–52, 54, 56) reported MMSE outcome, and the meta-analysis showed that that CHM can significantly improve the cognitive function compared to no intervention or placebo when assessed by MMSE (MD 1.96, 95% CI 1.41–2.50, 23 trials, 2156 participants, I^2^ = 89%, Figure 5). Nine trials (9, 15, 17, 20, 27, 36, 43, 46, 54) reported MoCA outcome, and the meta-analysis showed that CHM can significantly improve the cognitive function compared to no intervention or placebo when assessed by MoCA (MD 2.44, 95% CI 1.57–3.31, 728 participants, I^2^ = 81%, Figure 6). The heterogeneity was high. Therefore, we tried to conduct subgroup analyses in attempts to identify the difference in the effect. The results showed statistically significant differences when comparing trials with different CHM (test for subgroup difference: MMSE: *P* < 0.00001, I^2^ = 89.6%; MoCA: *P* = 0.05, I^2^ = 57.8%, Supplementary Figures 1, 2), trials with different causes of MCI (test for subgroup difference: MMSE: *P* < 0.00001, I^2^ = 89.1%; MoCA: *P* = 0.0002, I^2^ = 84.4%, Supplementary Figures 3, 4), and trials with different treatment duration (test for subgroup difference: MMSE: *P* = 0.06, I^2^ = 72.4%; MoCA: *P* < 0.00004, I^2^ = 92.1%, Supplementary Figures 5, 6). The subgroup analysis of different CHM showed that Ginko leaf tablets can significantly improve the cognitive function compared to no intervention or placebo when assessed by MMSE (MD 2.03, 95% CI 1.18–2.88, 1117 participants, I^2^ = 90%, Supplementary Figure 1) and MoCA (MD 3.11, 95% CI 1.90–4.33, 463 participants, I^2^ = 82%, Supplementary Figure 2). The sensitivity analysis by including only trials with relatively lower risk of bias found similar results (MD 1.87, 95% CI 1.22–2.52, 2 trials, 120 participants) with that including all trials (MD 1.96, 95% CI 1.41–2.50, 23 trials, 2,156 participants) showing robustness of the results on MMSE (Supplementary Figure 13).

#### ADL

**Figure 7:**
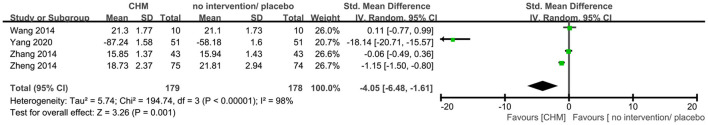
Forest plot of comparison: CHM vs. no intervention/placebo, outcome: ADL.

Four trials (20, 40, 41, 56) reported ADL outcome, of which one trial (56) used 0–100 ADL scale with higher score showing better results while the other three trials used the ADL scale with lower score showing better results. As ADL scales with differences in the direction have been used, we first multiplied the mean value of the trial set (56) by −1 to ensure the scales in the same direction, and then used SMD to synthesize data as recommended by Cochrane handbook. The meta-analysis showed that CHM significantly decreased the ADL score compared to no intervention and placebo (SMD −4.05, 95% CI −6.48 to −1.61, 357 participants, I^2^ = 100%, Figure 7).

#### CHM vs. Western Medicine (Co-intervention Was Allowed)

##### Cognitive Function

**Figure 8:**
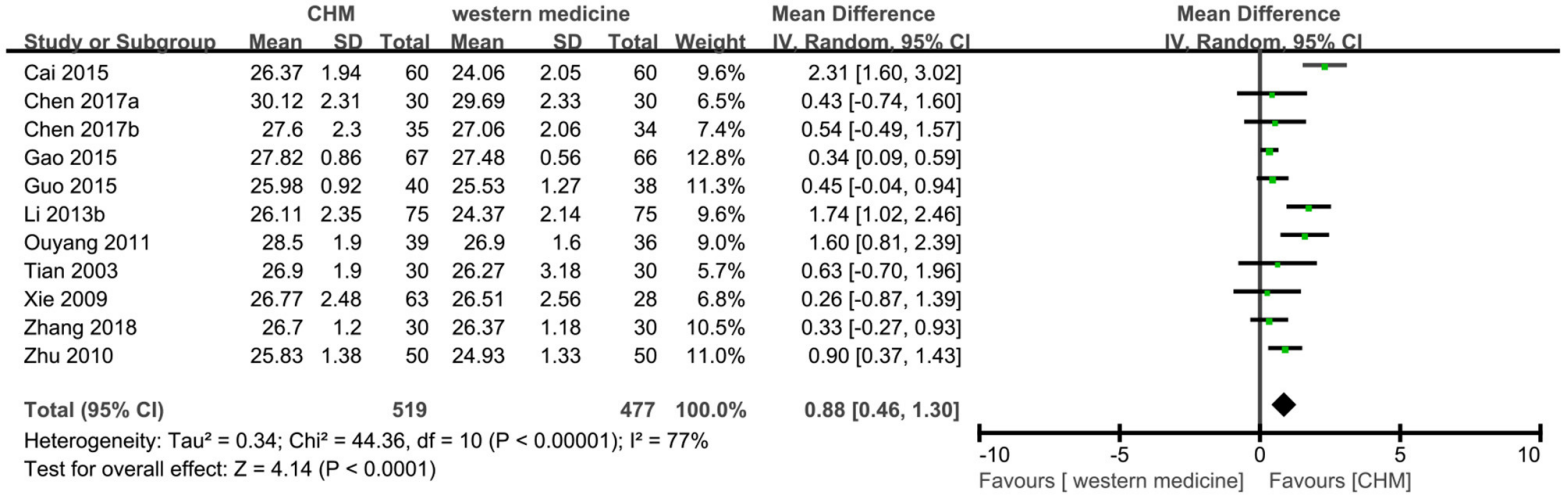
Forest plot of comparison: CHM vs. western medicine, outcome: MMSE.

Eleven trials (8, 12, 13, 18, 24, 26, 28, 31, 37, 39, 49) reported MMSE outcome, and the meta-analysis showed that TCM had a significant increase on the score of MMSE compared to western medicine (MD 0.88, 95% CI 0.46–1.30, 996 participants, I^2^ = 77%, Figure 8). Because of the high heterogeneity, the subgroup analyses were conducted according to different interventions and causes of MCI. The statistically significant subgroup difference was found when comparing different interventions (test for subgroup difference: *P* < 0.0001, I^2^ = 77.5%, Supplementary Figure 7), and when comparing different causes of MCI (test for subgroup difference: *P* < 0.0001, I^2^ = 82.9%, Supplementary Figure 8).

**Figure 9:**
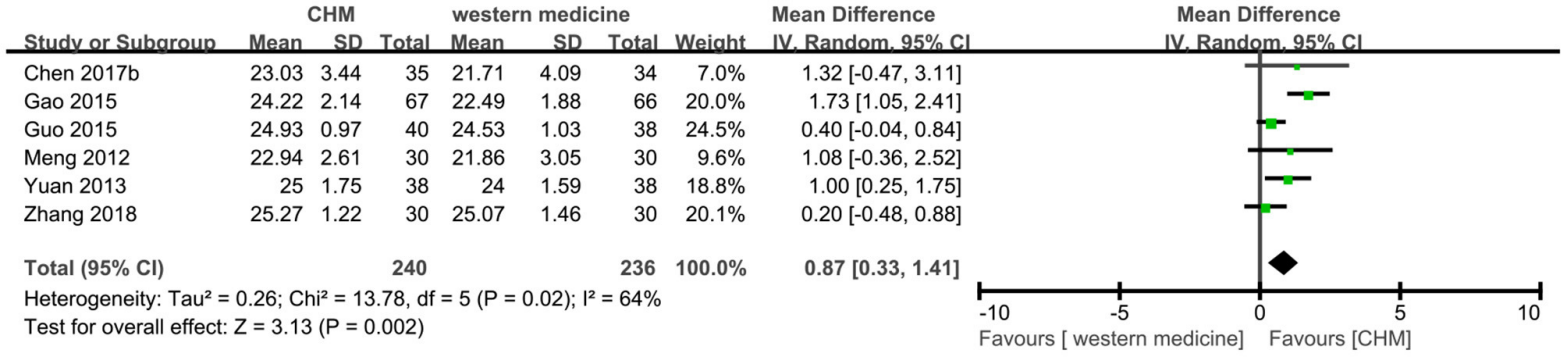
Forest plot of comparison: CHM vs. western medicine, outcome: MoCA.

Six trials (8, 13, 18, 22, 39, 47) reported the MoCA score, and the meta-analysis showed that CHM had a significant increase on the score of MoCA compared to western medicine (MD 0.87, 95% CI 0.33–1.41, I^2^ = 64%, Figure 9). Because of the high heterogeneity, the subgroup analyses were conducted according to different interventions and causes of MCI. The statistically significant subgroup difference was found when comparing different interventions (test for subgroup difference: *P* = 0.01, I^2^ = 69.6%, Supplementary Figure 9), and when comparing different causes of MCI (test for subgroup difference: *P* = 0.17, I^2^ = 42.8%, Supplementary Figure 10).

#### ADL

**Figure 10:**
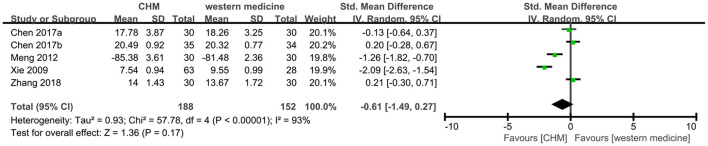
Forest plot of comparison: CHM vs. western medicine, outcome: ADL.

Five trials (8, 12, 13, 22, 28) reported this outcome, of which one trial (22) used ADL scale (higher score indicates better results) in the different direction with other scales (lower score indicates better results). We multiplied the mean value of 22 and used SMD model to synthesize the data. And the meta-analysis showed that CHM had no significant difference in the score of ADL compared to western medicine (SMD −0.61, 95% CI −1.49 to 0.27, I^2^ = 93%, Figure 10).

#### Adverse Events

Twenty-four trials reported adverse events, of which 13 trials reported that no adverse events ever occurred in both the CHM group and the control group. The remaining 11 trials found no significant difference between two groups on adverse events.

## Discussion

This review summarized current evidence on the comparative effectiveness of CHM, either individually tested or in combined remedies, for treatment of MCI, and finally identified 49 trials (48 finished trials and 1 protocol). This study found that CHM was better than no intervention or placebo (when co-intervention was allowed) in improving the number of participants who progressed to dementia and the score of MMSE, MoCA, ADL. And CHM was better than western medicine (when co-intervention was allowed) on improving the score of MMSE and MoCA but the significant difference was not found on the score of ADL. Considering the high heterogeneity, the subgroup analysis was conducted according to intervention, cause of MCI, and treatment duration.

In the subgroup analysis of intervention, we found that CHM Gingko leaf extracts were explored most. The combined effect from meta-analyses showed that Gingko leaf extracts as adjuvant therapy could slow down the progression to dementia, improve the MMSE score and MoCA score. Previous study showed that Gingko leaf extracts have been widely used in treating neuropsychiatric disorders (57). A previous systematic review published in 2016 (58) evaluated Ginkgo biloba in MCI and Alzheimer's diseases, and provided a similar result with our review stating that Ginkgo biloba in combination with conventional medicine is superior to conventional medicine alone in improving MMSE scores for people with MCI. The results of our study may provide support that Ginkgo leaf extracts can be considered for use in patients with MCI. However, still more qualified RCTs were needed to confirm the effect and optimize the use of Gingko leaf extracts. In the subgroup analysis of the cause of MCI, we found that CHM can improve the number of participants who progressed to dementia and the score of MMSE and MoCA in Parkinson related MCI patients compared to no intervention or placebo; CHM can improve the score of MMSE and MoCA of post-stroke and vascular MCI patients compared to no intervention or placebo; CHM can improve the score of MMSE, MoCA and ADL of post-stroke MCI patients compared to western medicine. However, more high-quality RCTs with large samples were needed to further confirm the effectiveness of CHM for the specific type of MCI. In the subgroup analysis of treatment duration, we found that CHM can improve the score of MMSE and MoCA in both subgroups, more than 6 months group and ≤ 6 months group.

Our review identified that the recommended outcomes by the guideline, including progression to dementia, reduction of ability to undertake daily activities, and quality of life (59), were infrequently assessed in the included trials than scales/ tools for cognition. Seven out of the included 45 trials have used a long-term endpoint progression to dementia as an outcome; eight trials have used scales of daily life activities; none of the trials has used quality of life. Oppositely, many different scales/ tools for cognitive function and global function have been used. The various scales/tools being used within the included trials limited the synthesis of data from different clinical trials. Moreover, the superiority of one scale/tool over another one has not been proved and should be explored in future (59). Besides, preservation of the patient's personality or the accessibility of disease information and health services should also be focused on future MCI studies from the voices of stakeholders (60). Outcome recommendations for dementia or cognitive function have been reported though (59–61), core outcome set specifically for MCI is still needed and should be developed.

As for methodological quality, future trials are recommended to be reported according to the CONSORT statement and its extension for herbal medicinal interventions (62, 63). Placebo-controlled design should be introduced. For trials comparing TCM with other western medicine, the double-dummy technique should be adopted to compare drugs with very different appearances to reduce observer and patients bias (64). The high risk of other bias was mainly shown in the funding and personnel team of the included trials. Six trials had only one author and we could not obtain any information of acknowledgment. Although the quality of RCTs cannot be decided by the number of the authors, for RCTs if conducted by only one author, the blinding was impossible. Four trials were suspected of sponsorship bias either because the authors were from the pharmaceutical companies or the companies fully or partially funded the trials. The previous review has suggested that industry-sponsored studies were biased in favor of the sponsor's products (65). The small sample size effect on publication bias should be cautioned as the included studies seemed to be not large, ranging from 48 to 245 participants. Sample size estimation is not available in most of the included studies, thereby whether the statistical power is enough seems to be unclear. Large sample size trials with strong power is warranted in future CHM studies for MCI.

There were similar systematic reviews previously published. One systematic review published in 2009 (6) explored CHM for MCI and age-associated memory impairment, and found the effects of the CHM was at least equivalent to piracetam on MMSE scores. Two systematic reviews by Lin et al. (4, 5) explored the effects of CHM for MCI by focusing either on MMSE and Alzheimer's disease assessment scale-cognitive subscale (4) or on MoCA (5). Our review was registered on PROSPERO and conducted a comprehensive up-to-date search of Chinese and English language databases to 2021 December. In this review, we focused on CHM, which is characterized by refined dosage forms and relative standardization and is approved by the State Administration for Market Regulation (66). To explore the benefits and harms of CHM, in this review, we focused on primary outcomes including the number of participants who progressed into dementia, cognitive function measured by accepted scales/tools, and adverse events.

There were some limitations in this review. Firstly, many included trials were judged to have the unclear risk of bias because we could not obtain enough information from the trial authors. Furthermore, there was certain clinical heterogeneity in the included RCTs because of causes of MCI, treatment duration.

## Conclusion

CHM, Ginko leaf extracts in particular, could help to prevent progression into dementia and to improve cognitive function and ability of daily living activities. However, due to the low quality of current trials, more qualified large-sample randomized controlled trials were needed to confirm the conclusion.

## Data Availability Statement

The original contributions presented in the study are included in the article/Supplementary Material, further inquiries can be directed to the corresponding authors.

## Author Contributions

NS was the project leader and initiated the study. YW and ZZ contributed to the conception of the study. NL provided the methodological guidance, trained the reviewers, and drafted the manuscript. SY, CL, LG, and SW searched the literature, collected the data, and evaluated the quality of the included studies. YC performed the meta-analysis and drafted the manuscript. All authors read and approved the final manuscript.

## Funding

The study was supported by National Key R&D Plan (2019YFC1712000) and National Science and Technology Major Project (2018ZX10101001-005).

## Conflict of Interest

The authors declare that the research was conducted in the absence of any commercial or financial relationships that could be construed as a potential conflict of interest.

## Publisher's Note

All claims expressed in this article are solely those of the authors and do not necessarily represent those of their affiliated organizations, or those of the publisher, the editors and the reviewers. Any product that may be evaluated in this article, or claim that may be made by its manufacturer, is not guaranteed or endorsed by the publisher.

## Abbreviations

AchE: Acetylcholinesterase
ADAS-Cog: Alzheimer's Disease Assessment Scale-Cognitive subscale
ADL: Activities of daily living scale
AE: Adverse events
AVLT: Auditory verbal learning test
BI: Barthel index
BNT: Boston naming test
C: Control
CACMS: China Academy of Chinese Medical Sciences
CDT: Clock drawing task
CGI- EI: Clinical global impression-efficacy index
CHD: Coronary heart disease
CHM: Chinese herbal medicine
CIs: confidence intervals
CNKI: China National Knowledge Infrastructure
CQVIP: Chongqing VIP
CRP: C-reactive protein
CVFT: Category verbal fluency test
DASS-21: 21-item Depression
Anxiety: Stress scale
D-KEFS: Delis-Keplan executive function system
DS: Digit span
DSR: Delayed story recall
EEG: Electroencephalogram
ERP P300: Event-related potentials P300
GDS: Geriatric depression scale
HAMD: Hamilton Depression Scale
HDL-C: High density liptein-cholesterol
I: Intervention
IADL: Instrumental activities of daily living scale
IBRCM: Institute of Basic Research in Clinical Medicine
LDL-C: Low density lipoprotein-cholesterol
MCI: mild cognitive impairment
MD: mean difference
MDA: Maloudialdehyde
MMSE: Mini-mental state examination
MoCA: Montreal Cognitive Assessment
MQ: Memory quotient
MRI: Magnetic resonance imaging
NHFPC: National Health and Family Planning Commission
NR: Not reported
RCFS: Rey complex figure test
RCTs: randomized clinical trials
ROCF: Rey-Osterrieth complex figure test
RR: risk ratio
RVR: Rapid verbal retrieve
SAMR: State Administration for Market Regulation
SAS: Self-rating anxiety scale
SCWT: Stroop color and word test
SDMT: Symbol digit modalities test
SDS: Self-rating depression scale
SMD: standard mean difference
STAI-X1: State-Trait anxiety inventory
SVD: Cerebral small vessel disease
TC: Total cholesterol
TCM: Traditional Chinese medicine
TG: Triglyceride
TMT: Trail-Making test
WMS: Wechsler memory scale.
