# Supplementary material for: Chinese Herbal Medicine for Mild Cognitive Impairment: A Systematic Review of Randomized Controlled Trials
Source: Front Neurol. 2022 Jun 30;13:903224. doi: 10.3389/fneur.2022.903224 (PMC9279622; doi:10.3389/fneur.2022.903224)
Supplement: Supplementary file 1 [file Data_Sheet_1.pdf]

Supplementary Files

1. Supplementary Tables

Supplementary Table 1

Searching strategy

Data sources	Search terms
PubMed	#1 Medicine, Chinese Traditional [Mesh]<br>#2 Traditional Chinese medicine [Title/ Abstract]<br>#3 Chinese herbal medicine [Title/ Abstract]<br>#4 Chinese material medica [Title/ Abstract]<br>#5 Chinese proprietary medicine [Title/ Abstract]<br>#6 Chinese patent medicine [Title/ Abstract]<br>#7 Gingko [Title/ Abstract]<br>#8 Congrongyizhi [Title/ Abstract]<br>#9 #1 OR #2 OR #3 OR #4 OR #5 OR #6 OR #7 OR #8<br>#10 Mild cognitive impairment [Title/ Abstract]<br>#11 MCI [Title/ Abstract]<br>#12 #10 OR #11<br>#13 RCT [All fields]<br>#14 random [All fields]<br>#15 randomized [All fields]<br>#16 randomised [All fields]<br>#17 #13 OR #14 OR #15 OR #16<br>#18 #9 AND #12 AND #17
Cochrane Library	#1 (Traditional Chinese medicine):ti,ab,kw OR (Chinese herbal medicine):ti,ab,kw OR (Chinese material medica):ti,ab,kw OR (Chinese proprietary medicine):ti,ab,kw OR (Chinese patent medicine):ti,ab,kw OR (Gingko):ti,ab,kw OR (Congrongyizhi):ti,ab,kw<br>#2 (Mild cognitive impairment):ti,ab,kw OR (MCI):ti,ab,kw<br>#3 (RCT) OR (random) OR (randomized) OR (randomised)<br>#4 #1 AND #2 AND #3
CNKI (Chinese)	#1 SU=("轻度认知障碍"+"轻度认知损害"+"轻度认知损伤"+"轻度认知缺损"+"轻度认知减退"+"轻度认知受损"+"轻度认知功能障碍"+"轻度认知功能损害"+"轻度认知功能损伤"+"轻度认知功能缺损"+"轻度认知功能减退" +"轻度认知功能受损")<br>#2 SU=("中药"+"草药"+"中草药"+"中成药"+"成药" +"复方"+"汤剂"+"方剂"+"中西医"+"中医")<br>#3 FT=("随机"+"盲法"+"安慰剂")<br>#4 #1 AND #2 AND #3
Wanfang Database	#1 主题:"轻度认知障碍"+主题:"轻度认知损害"+主题:"轻度认知损伤"+主题:"

(Chinese)	轻度认知缺损"+主题:"轻度认知减退" +主题:"轻度认知受损"+主题:"轻度认知功能障碍"+主题:"轻度认知功能损害"+主题:"轻度认知功能损伤"+主题:"轻度认知功能缺损"+主题:"轻度认知功能减退"+主题:"轻度认知功能受损"<br>#2 主题:"中药"+主题:"草药"+主题:"中草药"+主题:"中成药"+主题:"成药"+主题:"复方"+主题:"汤剂"+主题:"方剂"+主题:"中西医"+主题:"中医"<br>#3 全部:"随机"+全部:"盲法"+全部:"安慰剂"<br>#4 #1 AND #2 AND #3
Chongqing VIP (CQVIP) (Chinese)	#1 题名或关键词: 轻度认知功能障碍+mild cognitive impairment+slight cognition disfunction+轻度认知功能损害+轻度认知障碍+轻度认知功能损伤+轻度认知损害+轻度认知损伤+轻微认知功能损害+轻度认知缺损+轻度认知功能缺损+轻度认知减退+轻度认知功能减退+轻度认知受损+轻度认知功能受损<br>#2 题名或关键词: 中药+草药+中草药+中成药+成药+复方+汤剂+方剂+中西医+中医<br>#3 #1 AND #2
Sinomed (Chinese)	#1 "轻度认知障碍"[加权:扩展]<br>#2 "轻度认知障碍"[常用字段:智能] OR "轻度认知损害"[常用字段:智能] OR "轻度认知损伤"[常用字段:智能] OR "轻度认知缺损"[常用字段:智能] OR "轻度认知减退"[常用字段:智能] OR "轻度认知受损"[常用字段:智能] OR "轻度认知功能障碍"[常用字段:智能] OR "轻度认知功能损害"[常用字段:智能] OR "轻度认知功能损伤"[常用字段:智能] OR "轻度认知功能缺损"[常用字段:智能] OR "轻度认知功能减退"[常用字段:智能] OR "轻度认知功能受损"[常用字段:智能] OR "轻微认知功能损害"[常用字段:智能]<br>#3 (#1) OR (#2)<br>#4 "中药"[常用字段:智能] OR "草药"[常用字段:智能] OR "中草药"[常用字段:智能] OR "中成药"[常用字段:智能] OR "成药"[常用字段:智能] OR "复方"[常用字段:智能] OR "汤剂"[常用字段:智能] OR "方剂"[常用字段:智能] OR "中西医"[常用字段:智能] OR "中医"[常用字段:智能]<br>#5 (#3) AND (#4)<br>#6 "随机"[全部字段:智能] OR "盲法"[全部字段:智能] OR "安慰剂"[全部字段:智能]<br>#7 (#5) AND (#6)

Supplementary Table 2

The composition of Chinese herbal medicine explored in the included trials

**Chinese herbal medicine**	**Composition (Latin binomial name）**
Ginko leaf extracts	yín xìng yè (*Folium Ginkgo*)
Tongxinluo capsule	rén shēn (*Radix et Rhizoma Ginseng*); shuǐ zhì (*Hirudo*); quán xiē (*Scorpio*); chì sháo (*Radix Paeoniae Rubra*); chán tuì (*Periostracum Cicadae*); tǔ biē chóng (*Eupolyphaga seu Steleophaga*); wú gōng (*Scolopendra*); tán xiāng (*Lignum Santali Albi*); jiàng xiāng (*Lignum Dalbergiae Odoriferae*); rǔ xiāng (*Olibanum*); suān zǎo rén (fried) (*Semen Ziziphi Spinosae*); bīng piàn (*Borneolum Syntheticum*)
Liu Wei Di Huang pill	shú dì huáng (*Radix Rehmanniae Praeparata*); shān zhū yú (prepared) (*Fructus Corni*); mǔ dān pí (*Cortex Moutan*); shān yào (*Rhizoma Dioscoreae*); fú líng (*Poria*); zé xiè (*Rhizoma Alismatis*)
Yangxue Qingnao granules	dāng guī (*Radix Angelicae Sinensis*); chuān xiōng (*Rhizoma Chuanxiong*); bái sháo (*Radix Paeoniae Alba*); shú dì huáng; gōu téng (*Ramulus Uncariae Cum Uncis*); jī xuè téng (*Caulis Spatholobi*); xià kū cǎo (*Spica Prunellae*); jué míng zǐ (*Semen Cassiae*); zhēn zhū mǔ (*Concha Margaritiferae Usta*); yán hú suǒ (*Rhizoma Corydalis*); xì xīn (*Radix et Rhizoma Asari*)
Gui Ling Ji capsule	hóng shēn (*Radix et Rhizoma Ginseng Rubra*); lù róng (*Cornu Cervi Pantotrichum*); hǎi mǎ (*Hippocampus*); gǒu qǐ zǐ (*Fructus Lycii*); dīng xiāng (*Flos Caryophylli*); chuān shān jiǎ (*Squama Manitis*);què nǎo (*Passer montanus (Linnaeus)*); niú xī (*Radix Achyranthis Bidentatae*); suǒ yáng (*Herba Cynomorii*); shú dì huáng; bǔ gǔ zhī (*Fructus Psoraleae*); tù sī zǐ (*Semen Cuscutae*); dù zhòng (*Cortex Eucommiae*); shi yan (*Cyrtiospirifer sinensis (Graban.)*); ròu cōng róng (*Herba Cistanches*); gān cǎo (*Radix et Rhizoma Glycyrrhizae*); tiān dōng (*Radix Asparagi*); yín yáng huò (*Herba Epimedii*); dà qīng yán (*Halitum*); shā rén (Fructus Amomi); et al.

Astragalus injection	huáng qí (Radix Astragali)
Qizhi Tongluo capsule	shuǐ zhì; dì lóng (*Pheretima*); quán xiē; tǔ biē chóng; jiāng cán (*Bombyx Batryticatus*); bīng piàn; huáng qí; dān shēn (*Radix et Rhizoma Salviae Miltiorrhizae*); hóng huā (*Flos Carthami*); zé lán (*Herba Lycopi*); yù jīn (*Radix Curcumae*); dāng guī; jī xuè téng; rén shēn; dǎn nán xīng (*Arisaema cum Bile*); chì sháo; tiān má (*Rhizoma Gastrodiae*); jiāng huáng (*Rhizoma Curcumae Longae*); chuān xiōng; máo dōng qīng (*Radix Ilex Pubescens*); mài dōng (*Radix Ophiopogonis*); wǔ wèi zǐ (*Fructus Schisandrae Chinensis*); zhū yá zào (*Fructus Gleditsiae Abnormalis*); qiāng huó (*Rhizoma et Radix Notopterygii*); ròu guì (*Cortex Cinnamomi*); hé shǒu wū (*Radix Polygoni Multiflori*)
Tianmeng oral liquid	cì wǔ jiā (*Radix et Rhizoma seu Caulis Acanthopanacis Senticosi*); huáng jīng (*Rhizoma Polygonati*); can e (*Bombyx mori*); sāng shèn (*Fructus Mori*); dǎng shēn (*Radix Codonopsis*); huáng qí; shā rén; gǒu qǐ zǐ; shān zhā (*Fructus Crataegi*); shú dì huáng; yín yáng huò (prepared); chén pí (*Pericarpium Citri Reticulatae*); fú líng; mǎ qián zǐ (*Semen Strychni*)(prepared); fǎ bàn xià (*Rhizoma Pinelliae Praeparatum*); zé xiè; shān yào
Compound sea snake capsule	Hǎi Shé (*Pelamis platurus*), hǎi shēn (*Stichopus*); yuǎn zhì (*Radix Polygalae*); shí chāng pú (*Rhizoma Acori Tatarinowii*)
Compound Congrong Yizhi capsule (Bushen capsule)	hé shǒu wū; ròu cōng róng; dì lóng; lòu lú (*Radix Rhapontici*); hé yè (*Folium Nelumbinis*)
Jiannao Bushen	rén shēn; lù róng; gǒu shèn (*Peni et Testes Canitis*); ròu guì; Jīn Niú Cǎo (*Polygala arvensis Willd*);

pill	niú bàng zǐ (*Fructus Arctii*)(fried); jīn yīng zǐ (*Fructus Rosae Laevigatae*); dù zhòng (Charcoal); chuān niú xī (*Radix Cyathulae*); jīn yín huā (*Flos Lonicerae Japonicae*); lián qiào (*Fructus Forsythiae*); chán tuì; shān yào; yuǎn zhì (prepared with Licorice water); suān zǎo rén; shā rén; dāng guī; lóng gǔ (*Fossilia Ossis Mastodi*)(Calcined); mǔ lì (*Concha Ostreae*)(Calcined); fú líng; bái zhú (*Rhizoma Atractylodis Macrocephalae*)(fried with bran); guì zhī (*Ramulus Cinnamomi*); gān cǎo; bái sháo (fried with liqueur); dòu kòu (Fructus Amomi Rotundus)
Shenzhiling oral liquid	dǎng shēn; guì zhī; bái sháo; zhì gān cǎo (*Radix et Rhizoma Glycyrrhizae Praeparata cum Melle*); fú líng; gān jiāng (*Rhizoma Zingiberis*); yuǎn zhì (prepared); shí chāng pú; lóng gǔ; mǔ lì
Jinsiwei pill	ròu cōng róng; gǒu qǐ zǐ; shí chāng pú; yuǎn zhì; chuān xiōng, et al.
Tianzhi granules	tiān má; gōu téng; shí jué míng (*Concha Haliotidis*); dù zhòng; sāng jì shēng (*Herba Taxilli*); fú shén (*Sclerotium Poriae Pararadicis*); shǒu wū téng (*Caulis Polygoni Multiflori*); huái huā (*Flos Sophorae*); zhī zǐ (*Fructus Gardeniae*); huáng qín (*Radix Scutellariae*); chuān niú xī; yì mǔ cǎo (*Herba Leonuri*)
Xuanyunning tablet	zé xiè; bái zhú; fú líng; fǎ bàn xià; nǚ zhēn zǐ (*Fructus Ligustri Lucidi*); mò hàn lián (*Herba Ecliptae*); jú huā (*Flos Chrysanthemi*); niú xī; chén pí; gān cǎo
Fufang Huonao Shu	Zhū Nǎo (*Sus scrofa domestica Brisson*); wǔ wèi zǐ; mài dōng; rén shēn; gǒu qǐ zǐ; dì huáng (*Radix Rehmanniae*); dān shēn
Yindanxinnaotong soft capsule	yín xìng yè; dān shēn; dēng zhǎn xì xīn (Herba Erigerontis); sān qī (Radix et Rhizoma Notoginseng); shān zhā; jiǎo gǔ lán (Rhizoma seu Herba Gynostemmatis Pentaphylli); dà suàn (Bulbus Allii); tiān rán bīng piàn (Borneolum)
Shuganjieyu	guàn yè jīn sī táo (Herba Hyperici Perforati); cì wǔ jiā

capsule	
Nao Li Bao pills	yuǎn zhì; dì huáng; wǔ wèi zǐ; dì gǔ pí (Cortex Lycii); tù sī zǐ; fú líng; shí chāng pú; chuān xiōng
Huan Shao capsules	ròu cōng róng; bā jǐ tiān (Radix Morindae Officinalis); xiǎo huí xiāng (Fructus Foeniculi); shú dì huáng; gǒu qǐ zǐ; dù zhòng; niú xī; fú líng; shān yào; shān zhū yú; wǔ wèi zǐ; yuǎn zhì; shí chāng pú; dà zǎo (Fructus Jujubae)
Sailuotong	rén shēn; yín xìng yè; xī hóng huā (Stigma Croci)
Memo	yín xìng yè; rén shēn; feng wang jiang (*Apis cerana Fabr/ Apis mellifera L.*)
Xinnaoning capsule	yín xìng yè; dān shēn; dà guǒ mù jiāng zǐ (Litsea lancilimba Merr.); xiǎo yè huáng yáng (Buxus sinica var. parvifolia M. Cheng); xiè bái (Bulbus Allii Macrostemi)
Quan Tian Ma capsule	tiān má

Supplementary Table 3

Detailed product information of Chinese herbal medicine explored in the included trials

Study ID	Chinese herbal medicine	Product name	Manufacturer of the product
Dong 2015	Gingko leaf extracts	SiTaiLong	Shanghai Xingling Technology Pharmaceutical
Han 2016	Gingko leaf extracts	SiTaiLong	Shanghai Xingling Technology Pharmaceutical
Li 2013a	Gingko leaf extracts	YiKangNing	Yangzi River Pharmaceutical Group
Wang 2012	Gingko leaf extracts	SiTaiLong	Shanghai Xingling Technology Pharmaceutical Co., Ltd
Xia 2013	Gingko leaf extracts	DaNaiKang	Beaufour Ipsen Industrie
Xiao 2011	Gingko leaf extracts	SiTaiLong	Shanghai Xingling Technology Pharmaceutical
Zhang 2013	Gingko leaf extracts	JinNaDuo	Dr. Willmar Schwabe GmbH & Co.KG
Zhang 2014	Gingko leaf extracts	-	-
Zhang 2015a	Extract of Ginkgo Biloba Leaves Injection	XingXue	ZBD Pharmaceutical
He 2014	Gingko leaf extracts	-	-
Su 2015	Gingko leaf extracts	-	Yiling Pharmaceutical
Zhang 2016	Gingko leaf extracts	-	-
Jia 2011	Tongxinluo	-	Yiling Pharmaceutical
Ye 2013	Tongxinluo	-	-
Yu 2017	Fufang Haishe capsule	XiEnKai	Hacon Pharmaceutical
Liu 2008	Yangxue Qingnao granules	-	-
Zhao 2011	Yangxue Qingnao granules	-	-
Lan 2015	Qizhi Tongluo capsule	-	-
Lin 2009	Astragalus injection	-	Chengdu Diao Pharmaceutical Co., Ltd
Wu 2017	Tianmeng oral liquid	-	Rongchang Pharmaceuticals
Zheng 2014	Yindanxinnaotong soft capsule	-	Guizhou Bailing Enterprise Group Pharmaceutical
Yakoot 2013	Memo capsule	-	-
Li 2009	Quan Tian Ma capsule	-	Eakang Pharmaceutical
Huang 2015	Liuwei Dihuang pills	NingAnTang	Hubei Ruihua Pharmaceutical
Wang 2014	Compound Congrong Yizhi capsule	-	-
Zhang 2010	Compound Congrong Yizhi capsule	-	-
Yang 2020	Yinxing Tongzhi tablets	SiTaiLong	Shanghai Xingling Technology Pharmaceutical Co., Ltd
Chen 2017a	Shenzhiling oral liquid	-	Wohua Pharmaceutical

Gao 2015	Compound Congrong Yizhi capsule	-	Liaoyuan Yulong Yadong Pharmaceutical
Ouyang 2011	Jiannao Bushen pill	-	Tsinghuawei Pharmaceutical
Xie 2009	Tianzhi granules	-	Henan Wanxi Pharmaceutical
Chen 2017b	Compound Congrong Yizhi capsule	-	Liaoyuan Yulong Yadong Pharmaceutical
Tian 2003	Jinsiwei pill	-	Henan Wanxi Pharmaceutical
Zhang 2018	Fufang Huonao Shu	-	Jilin Province Huinan Changlong Biochemical Pharmaceutical
Zhu 2010	Xuanyunning tablets	-	Sanjin Group Guilin Sanjin Biopharmaceuticals
Li 2013b	Ginkgo tablets	YiKangNing	Yangzi River Pharmaceutical Group
Cai 2015	Shuganjieyu capsule	-	Kanghong Pharmaceutical
Guo 2015	Xinnaoning capsule	-	Guizhou Antai Pharmaceutical
Meng 2012	Compound Congrong Yizhi capsule	-	Liaoyuan Yulong Yadong Pharmaceutical
Yuan 2013	Huan Shao capsules	-	Chongqing Sanxia Yunhai Pharmaceutical
Dong 2017	Gui Ling Ji capsule	-	GuangYuYuan Pharmaceutical
Zheng 2016	Ginkgo biloba extract tablet	-	Beijing Sihuan Pharmaceutical
Zhu 2002	Nao Li Bao pills	-	Henan Wanxi Pharmaceutical
Zhang 2015b	Compound Congrong Yizhi	-	Liaoyuan Yulong Yadong Pharmaceutical
Gavrilova 2014	Ebb 761 (Ginkgo biloba extracts tablets)	-	-
Liu 2020	Zhongfeng Huichun tablet, 5 tablets	Zhongfeng Huichun tablet Yuzhou Yaowang	Henan Yuzhou Yaowang Pharmaceutical
Guo 2020	Jinlida granules	-	Yiling Pharmaceutical
Gschwind 2017	GBE 1370	-	Symfona® forte

Supplementary Figures

Study or Subgroup	CHM Mean	CHM SD	CHM Total	no intervention/ placebo Mean	no intervention/ placebo SD	no intervention/ placebo Total	Weight	Mean Difference IV, Random, 95% CI
**1.2.1 Ginko leaf extracts**								
Dong 2015	26.41	2.47	57	22.75	2.34	57	5.2%	3.66 [2.78, 4.54]
Han 2016	26.41	3.79	60	22.55	3.54	60	4.6%	3.86 [2.55, 5.17]
Li 2013a	28.17	2.46	35	25.34	3.21	34	4.5%	2.83 [1.48, 4.18]
Wang 2012	23.84	1.97	45	22.93	2.03	42	5.3%	0.91 [0.07, 1.75]
Xia 2013	27.81	2.08	123	25.42	1.75	122	5.6%	2.39 [1.91, 2.87]
Xiao 2011	27.62	2.18	58	25.27	3.18	55	5.0%	2.35 [1.34, 3.36]
Zhang 2013	26.58	2.12	43	24.62	3.51	40	4.7%	1.96 [0.70, 3.22]
Zhang 2014	27.83	1.16	43	27.08	1.29	43	5.6%	0.75 [0.23, 1.27]
Zhang 2015a	25.17	3.02	100	25.08	1.55	100	5.5%	0.09 [-0.58, 0.76]
**Subtotal (95% CI)**			**564**			**553**	**46.0%**	**2.03 [1.18, 2.88]**
Heterogeneity: Tau² = 1.45; Chi² = 80.43, df = 8 (P < 0.00001); I² = 90%								
Test for overall effect: Z = 4.68 (P < 0.00001)								
**1.2.2 Compound Congrong Yizhi capsule**								
Wang 2014	27.7	1.42	10	27.1	0.88	10	5.0%	0.60 [-0.44, 1.64]
Zhang 2016	26.68	3.58	30	25.35	3.01	30	4.1%	1.33 [-0.34, 3.00]
**Subtotal (95% CI)**			**40**			**40**	**9.1%**	**0.80 [-0.08, 1.68]**
Heterogeneity: Tau² = 0.00; Chi² = 0.53, df = 1 (P = 0.47); I² = 0%								
Test for overall effect: Z = 1.79 (P = 0.07)								
**1.2.3 Yangxue Qingnao granules**								
Liu 2008	26.38	3.05	24	24.18	5.01	21	3.0%	2.20 [-0.27, 4.67]
Zhao 2011	26.33	3.14	35	25.46	5.01	35	3.7%	0.87 [-1.09, 2.83]
**Subtotal (95% CI)**			**59**			**56**	**6.7%**	**1.38 [-0.15, 2.92]**
Heterogeneity: Tau² = 0.00; Chi² = 0.69, df = 1 (P = 0.41); I² = 0%								
Test for overall effect: Z = 1.77 (P = 0.08)								
**1.2.4 Qizhi Tongluo capsule**								
Lan 2015	21.28	3.21	30	17.67	2.88	30	4.3%	3.61 [2.07, 5.15]
**Subtotal (95% CI)**			**30**			**30**	**4.3%**	**3.61 [2.07, 5.15]**
Heterogeneity: Not applicable								
Test for overall effect: Z = 4.58 (P < 0.00001)								
**1.2.5 Astragalus injection**								
Lin 2009	27.53	1.98	30	26.27	2.17	29	5.0%	1.26 [0.20, 2.32]
**Subtotal (95% CI)**			**30**			**29**	**5.0%**	**1.26 [0.20, 2.32]**
Heterogeneity: Not applicable								
Test for overall effect: Z = 2.33 (P = 0.02)								
**1.2.6 Tianmeng oral liquid**								
Wu 2017	28.88	1.01	53	24.94	1.15	49	5.7%	3.94 [3.52, 4.36]
**Subtotal (95% CI)**			**53**			**49**	**5.7%**	**3.94 [3.52, 4.36]**
Heterogeneity: Not applicable								
Test for overall effect: Z = 18.32 (P < 0.00001)								
**1.2.7 Tongxinluo**								
Ye 2013	28.75	4.24	30	26.68	3.42	33	3.7%	2.07 [0.16, 3.98]
**Subtotal (95% CI)**			**30**			**33**	**3.7%**	**2.07 [0.16, 3.98]**
Heterogeneity: Not applicable								
Test for overall effect: Z = 2.12 (P = 0.03)								
**1.2.8 Fufang Haishe capsule versus no intervention**								
Yu 2017	25.52	1.07	48	24.75	1.49	45	5.6%	0.77 [0.24, 1.30]
**Subtotal (95% CI)**			**48**			**45**	**5.6%**	**0.77 [0.24, 1.30]**
Heterogeneity: Not applicable								
Test for overall effect: Z = 2.85 (P = 0.004)								
**1.2.9 Yindanxinnaotong soft capsule**								
Zheng 2014	27.76	1.88	75	25.25	3.68	74	5.1%	2.51 [1.57, 3.45]
**Subtotal (95% CI)**			**75**			**74**	**5.1%**	**2.51 [1.57, 3.45]**
Heterogeneity: Not applicable								
Test for overall effect: Z = 5.23 (P < 0.00001)								
**1.2.10 Memo capsule**								
Yakoot 2013	26.97	1.38	30	25	1.41	30	5.4%	1.97 [1.26, 2.68]
**Subtotal (95% CI)**			**30**			**30**	**5.4%**	**1.97 [1.26, 2.68]**
Heterogeneity: Not applicable								
Test for overall effect: Z = 5.47 (P < 0.00001)								
**1.2.11 Quan Tian Ma capsule**								
Li 2009	25.78	3.15	30	23.8	4.91	30	3.5%	1.98 [-0.11, 4.07]
**Subtotal (95% CI)**			**30**			**30**	**3.5%**	**1.98 [-0.11, 4.07]**
Heterogeneity: Not applicable								
Test for overall effect: Z = 1.86 (P = 0.06)								
**Total (95% CI)**			**989**			**969**	**100.0%**	**1.98 [1.37, 2.60]**
Heterogeneity: Tau² = 1.68; Chi² = 204.33, df = 20 (P < 0.00001); I² = 90%								
Test for overall effect: Z = 6.33 (P < 0.00001)								
Test for subgroup differences: Chi² = 110.67, df = 10 (P < 0.00001), I² = 91.0%								

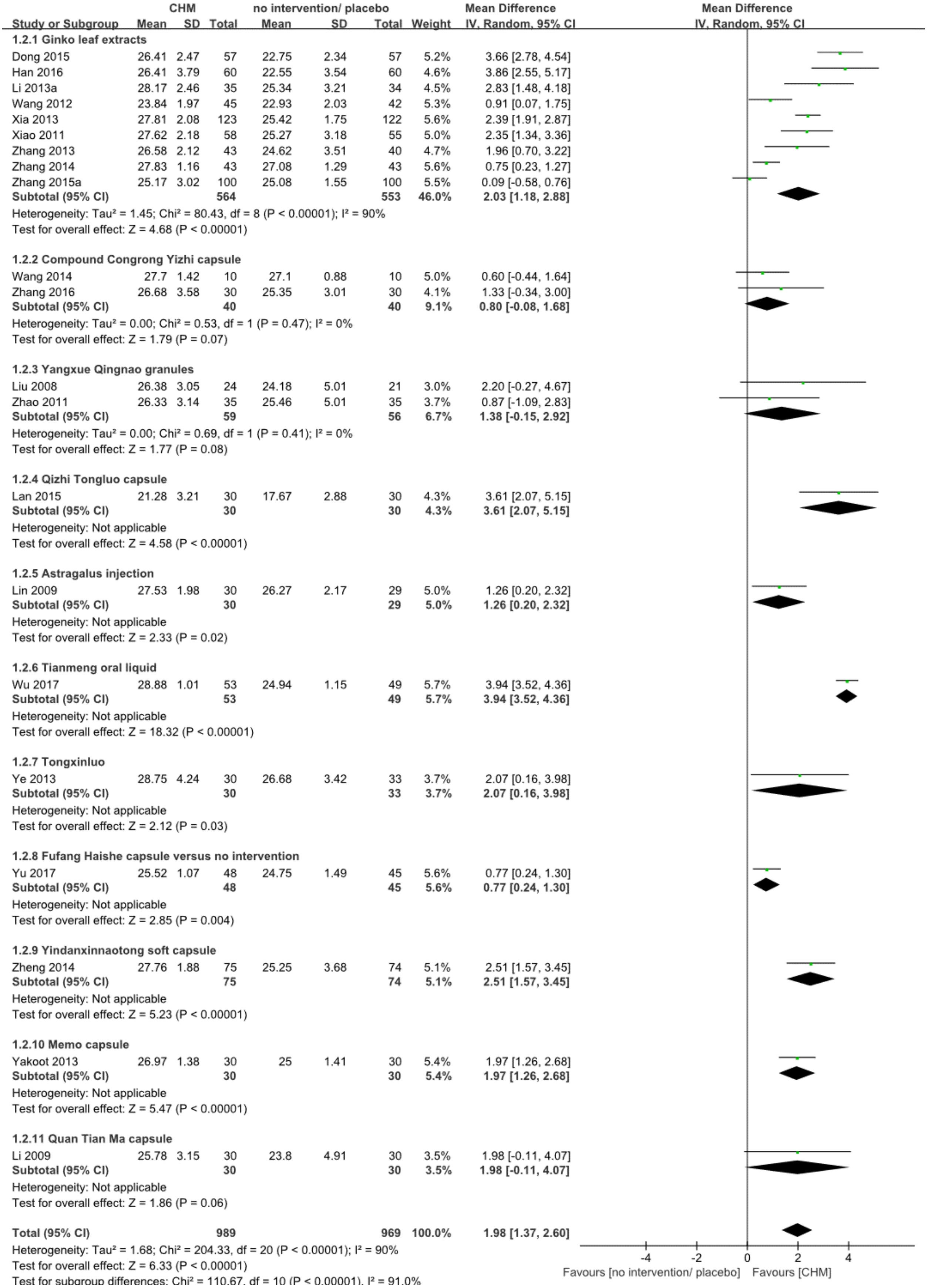

Supplementary Fig. 1. Forest plot of subgroup analyses of comparing trials with different Chinese herbal medicine and no intervention/ placebo, outcome: MMSE

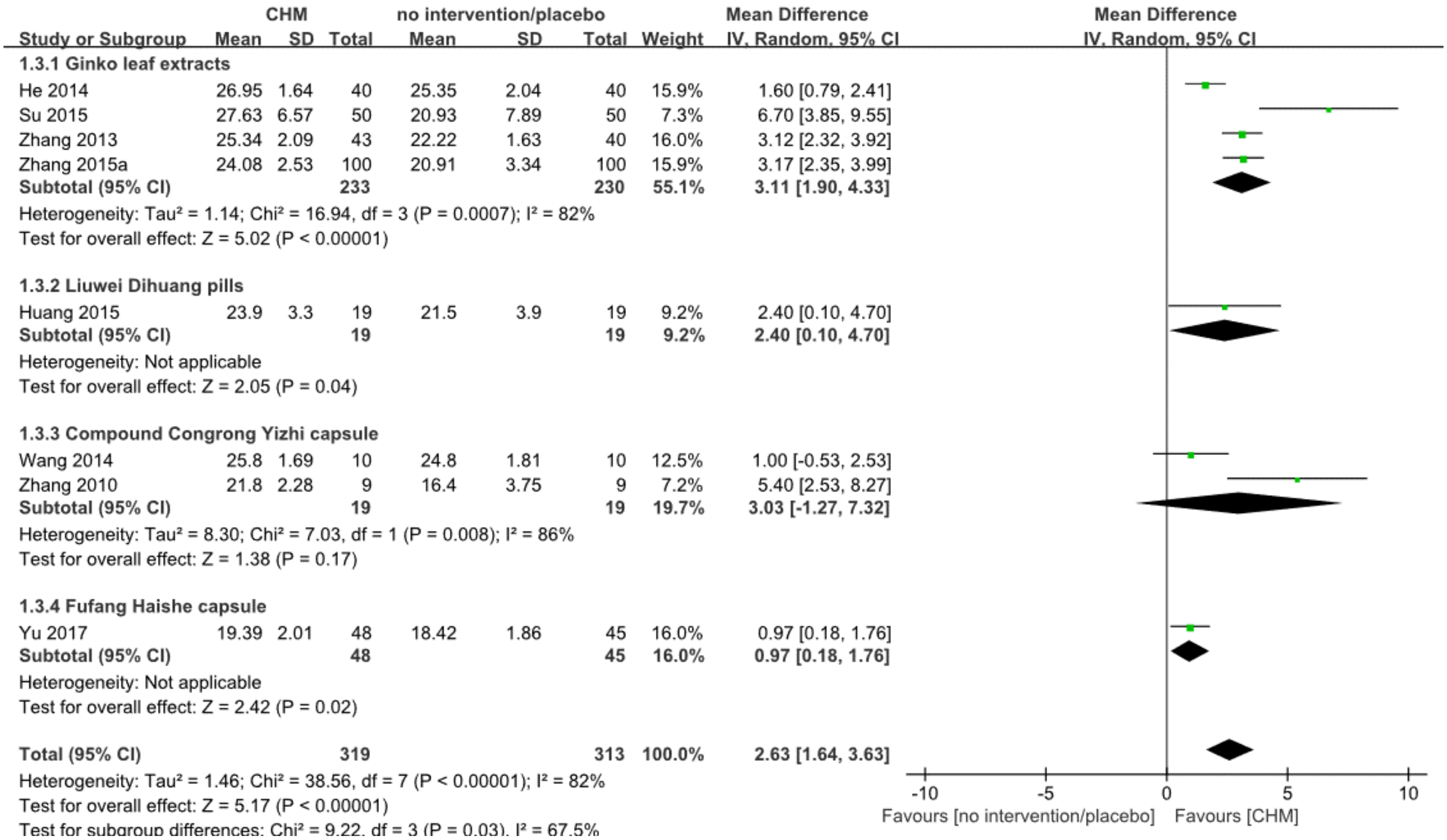

Supplementary Fig. 2. Forest plot of subgroup analyses of comparing trials with different Chinese herbal medicine and no intervention/ placebo, outcome: MoCA

Study or Subgroup	CHM Mean	CHM SD	CHM Total	no intervention/placebo Mean	no intervention/placebo SD	no intervention/placebo Total	Weight	Mean Difference IV, Fixed, 95% CI
**1.6.1 MCI without causes reported**								
Dong 2015	26.41	2.47	57	22.75	2.34	57	4.0%	3.66 [2.78, 4.54]
Li 2009	25.78	3.15	30	23.87	4.91	30	0.7%	1.91 [-0.18, 4.00]
Li 2013a	28.17	2.46	35	25.34	3.21	34	1.7%	2.83 [1.48, 4.18]
Wang 2012	23.84	1.97	45	22.93	2.03	42	4.4%	0.91 [0.07, 1.75]
Wu 2017	28.88	1.01	53	24.94	1.15	49	17.6%	3.94 [3.52, 4.36]
Xia 2013	27.81	2.08	123	25.42	1.75	122	13.5%	2.39 [1.91, 2.87]
Xiao 2011	27.62	2.18	58	25.27	3.18	55	3.1%	2.35 [1.34, 3.36]
Yakoot 2013	26.97	1.38	30	25	1.41	30	6.3%	1.97 [1.26, 2.68]
Ye 2013	28.75	4.24	30	26.68	3.42	33	0.9%	2.07 [0.16, 3.98]
Yu 2017	25.52	1.07	48	24.75	1.49	45	11.1%	0.77 [0.24, 1.30]
Zhang 2015a	25.17	3.02	100	25.08	1.55	100	7.1%	0.09 [-0.58, 0.76]
Zhang 2016	26.33	3.14	35	25.46	5.01	35	0.8%	0.87 [-1.09, 2.83]
Zheng 2014	27.76	1.88	75	25.25	3.68	74	3.5%	2.51 [1.57, 3.45]
**Subtotal (95% CI)**			**719**			**706**	**74.5%**	**2.23 [2.03, 2.44]**
Heterogeneity: Chi² = 155.70, df = 12 (P < 0.00001); I² = 92%								
Test for overall effect: Z = 21.37 (P < 0.00001)								
**1.6.4 vascular MCI**								
He 2014	26.15	2.23	40	25.35	2.04	40	3.6%	0.80 [-0.14, 1.74]
Lan 2015	21.28	3.21	30	17.67	2.88	30	1.3%	3.61 [2.07, 5.15]
Lin 2009	27.53	1.98	30	26.27	2.17	29	2.8%	1.26 [0.20, 2.32]
Liu 2008	26.38	3.05	24	24.18	5.01	21	0.5%	2.20 [-0.27, 4.67]
Wang 2014	27.7	1.42	10	27.1	0.88	10	2.9%	0.60 [-0.44, 1.64]
Zhang 2014	27.83	1.16	43	27.08	1.29	43	11.6%	0.75 [0.23, 1.27]
Zhao 2011	26.33	3.14	35	25.46	5.01	35	0.8%	0.87 [-1.09, 2.83]
**Subtotal (95% CI)**			**212**			**208**	**23.5%**	**0.99 [0.63, 1.36]**
Heterogeneity: Chi² = 13.79, df = 6 (P = 0.03); I² = 56%								
Test for overall effect: Z = 5.35 (P < 0.00001)								
**1.6.5 parkinson related MCI**								
Zhang 2013	26.58	2.12	43	24.62	3.51	40	2.0%	1.96 [0.70, 3.22]
**Subtotal (95% CI)**			**43**			**40**	**2.0%**	**1.96 [0.70, 3.22]**
Heterogeneity: Not applicable								
Test for overall effect: Z = 3.05 (P = 0.002)								
**Total (95% CI)**			**974**			**954**	**100.0%**	**1.94 [1.76, 2.11]**
Heterogeneity: Chi² = 203.10, df = 20 (P < 0.00001); I² = 90%								
Test for overall effect: Z = 21.47 (P < 0.00001)								
Test for subgroup differences: Chi² = 33.62, df = 2 (P < 0.00001), I² = 94.1%								

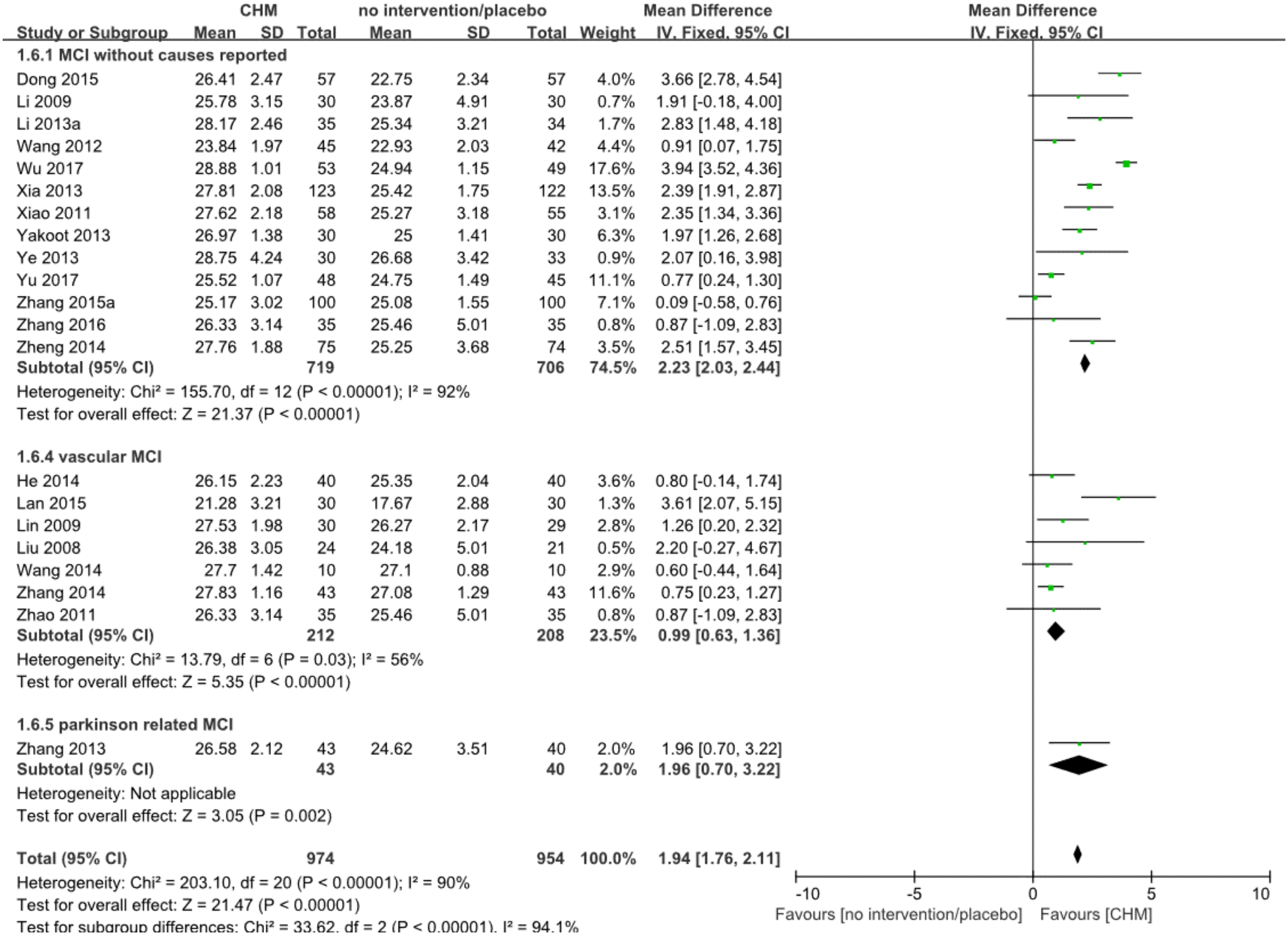

Supplementary Fig. 3. Forest plot of subgroup analyses of comparing trials with different causes of MCI of Chinese herbal medicine versus no intervention/ placebo, outcome: MMSE

Study or Subgroup	CHM Mean	CHM SD	CHM Total	no intervention/placebo Mean	no intervention/placebo SD	no intervention/placebo Total	Weight	Mean Difference IV, Fixed, 95% CI
**1.7.1 MCI without causes reported**								
Huang 2015	23.9	3.3	19	21.5	3.9	19	3.4%	2.40 [0.10, 4.70]
Su 2015	27.63	6.57	50	20.93	7.89	50	2.2%	6.70 [3.85, 9.55]
Zhang 2015a	24.08	2.53	100	20.91	3.34	100	26.7%	3.17 [2.35, 3.99]
**Subtotal (95% CI)**			**169**			**169**	**32.4%**	**3.33 [2.59, 4.08]**
Heterogeneity: Chi² = 6.16, df = 2 (P = 0.05); I² = 68%								
Test for overall effect: Z = 8.75 (P < 0.00001)								
**1.7.2 vascular MCI**								
He 2014	26.95	1.64	40	25.35	2.04	40	27.4%	1.60 [0.79, 2.41]
Wang 2014	25.8	1.69	10	24.8	1.35	10	10.0%	1.00 [-0.34, 2.34]
Zhang 2010	21.8	2.28	9	16.4	3.75	9	2.2%	5.40 [2.53, 8.27]
**Subtotal (95% CI)**			**59**			**59**	**39.7%**	**1.66 [0.98, 2.33]**
Heterogeneity: Chi² = 7.49, df = 2 (P = 0.02); I² = 73%								
Test for overall effect: Z = 4.82 (P < 0.00001)								
**1.7.4 parkinson related MCI**								
Zhang 2013	25.34	2.09	43	22.22	1.63	40	28.0%	3.12 [2.32, 3.92]
**Subtotal (95% CI)**			**43**			**40**	**28.0%**	**3.12 [2.32, 3.92]**
Heterogeneity: Not applicable								
Test for overall effect: Z = 7.61 (P < 0.00001)								
**Total (95% CI)**			**271**			**268**	**100.0%**	**2.61 [2.18, 3.03]**
Heterogeneity: Chi² = 26.43, df = 6 (P = 0.0002); I² = 77%								
Test for overall effect: Z = 12.04 (P < 0.00001)								
Test for subgroup differences: Chi² = 12.78, df = 2 (P = 0.002), I² = 84.4%								

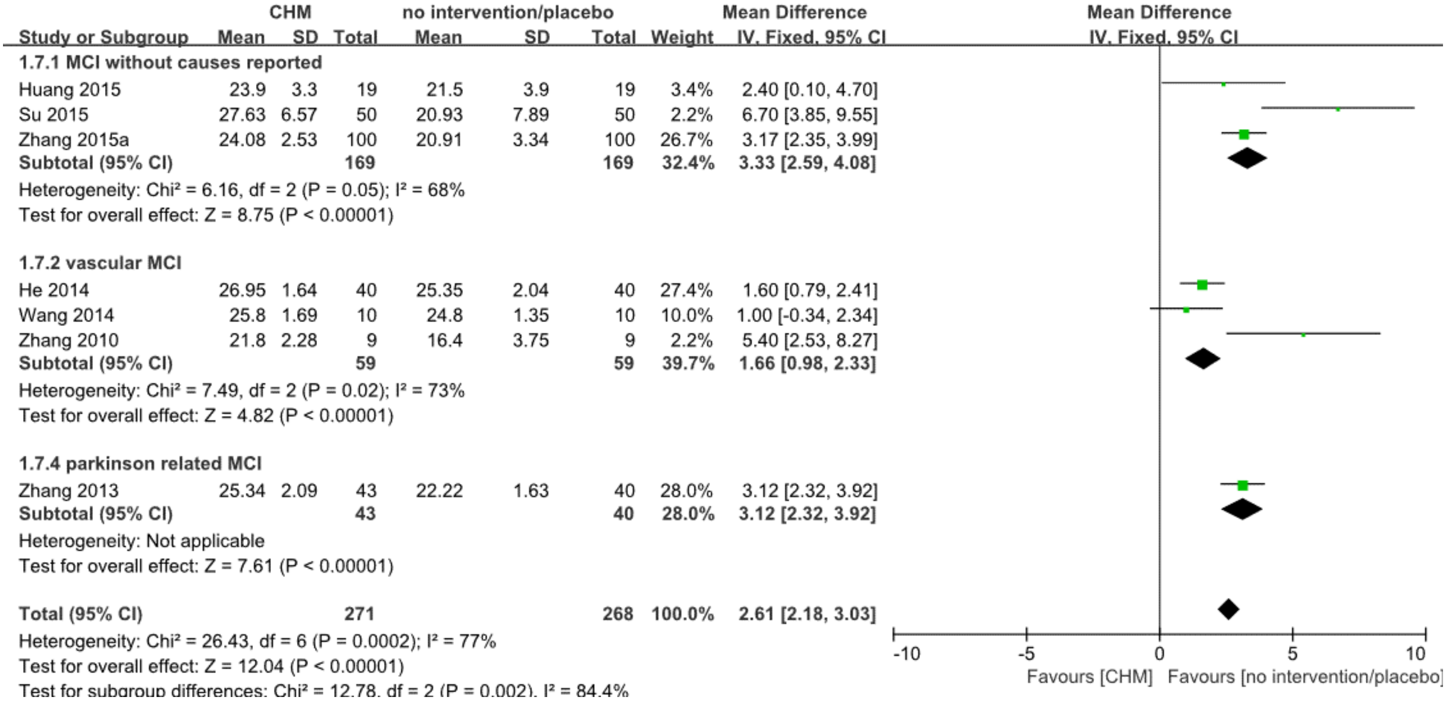

Supplementary Fig. 4. Forest plot of subgroup analyses of comparing trials with different causes of MCI of Chinese herbal medicine versus no intervention/ placebo, outcome: MoCA

Study or Subgroup	CHM Mean	CHM SD	CHM Total	no intervention/placebo Mean	no intervention/placebo SD	no intervention/placebo Total	Weight	Mean Difference IV, Fixed, 95% CI
**1.10.1 <=6m**								
Dong 2015	26.41	2.47	57	22.75	2.34	57	3.5%	3.66 [2.78, 4.54]
Guo 2020	26.98	1.85	48	25.37	1.96	48	4.7%	1.61 [0.85, 2.37]
He 2014	26.15	2.23	4	25.35	2.04	40	0.5%	0.80 [-1.47, 3.07]
Lan 2015	21.28	3.21	30	17.67	2.88	30	1.1%	3.61 [2.07, 5.15]
Li 2009	25.78	3.15	30	23.87	4.91	30	0.6%	1.91 [-0.18, 4.00]
Li 2013a	28.17	2.46	35	25.34	3.21	34	1.5%	2.83 [1.48, 4.18]
Lin 2009	27.53	1.98	30	26.27	2.17	29	2.4%	1.26 [0.20, 2.32]
Liu 2008	26.38	3.05	24	24.18	5.01	21	0.4%	2.20 [-0.27, 4.67]
Wang 2014	27.7	1.42	10	27.1	0.88	10	2.5%	0.60 [-0.44, 1.64]
Wu 2017	28.88	1.01	53	24.94	1.15	49	15.3%	3.94 [3.52, 4.36]
Yakoot 2013	26.97	1.38	30	25	1.41	30	5.4%	1.97 [1.26, 2.68]
Yang 2020	26.91	1.49	51	25.06	1.26	51	9.5%	1.85 [1.31, 2.39]
Ye 2013	28.75	4.24	30	26.68	3.42	33	0.7%	2.07 [0.16, 3.98]
Zhang 2013	26.58	2.12	43	24.62	3.51	40	1.7%	1.96 [0.70, 3.22]
Zhang 2014	27.83	1.16	43	27.08	1.29	43	10.1%	0.75 [0.23, 1.27]
Zhang 2015a	25.17	3.02	100	25.08	1.55	100	6.1%	0.09 [-0.58, 0.76]
Zhao 2011	26.33	3.14	35	25.46	5.01	35	0.7%	0.87 [-1.09, 2.83]
Zheng 2014	27.76	1.88	75	25.25	3.68	74	3.1%	2.51 [1.57, 3.45]
**Subtotal (95% CI)**			**728**			**754**	**69.9%**	**2.08 [1.88, 2.28]**
Heterogeneity: Chi² = 167.67, df = 17 (P < 0.00001); I² = 90%								
Test for overall effect: Z = 20.69 (P < 0.00001)								
**1.10.2 >6m**								
Han 2016	26.41	3.79	60	22.55	3.54	60	1.6%	3.86 [2.55, 5.17]
Wang 2012	23.84	1.97	45	22.93	2.03	42	3.8%	0.91 [0.07, 1.75]
Xia 2013	27.81	2.08	123	25.42	1.75	122	11.7%	2.39 [1.91, 2.87]
Xiao 2011	27.62	2.18	58	25.27	3.18	55	2.7%	2.35 [1.34, 3.36]
Yu 2017	25.52	1.07	48	24.75	1.49	45	9.7%	0.77 [0.24, 1.30]
Zhang 2016	26.68	3.58	30	25.46	5.01	35	0.6%	1.22 [-0.88, 3.32]
**Subtotal (95% CI)**			**364**			**359**	**30.1%**	**1.73 [1.43, 2.03]**
Heterogeneity: Chi² = 35.26, df = 5 (P < 0.00001); I² = 86%								
Test for overall effect: Z = 11.29 (P < 0.00001)								
**Total (95% CI)**			**1092**			**1113**	**100.0%**	**1.97 [1.81, 2.14]**
Heterogeneity: Chi² = 206.56, df = 23 (P < 0.00001); I² = 89%								
Test for overall effect: Z = 23.50 (P < 0.00001)								
Test for subgroup differences: Chi² = 3.63, df = 1 (P = 0.06), I² = 72.4%								

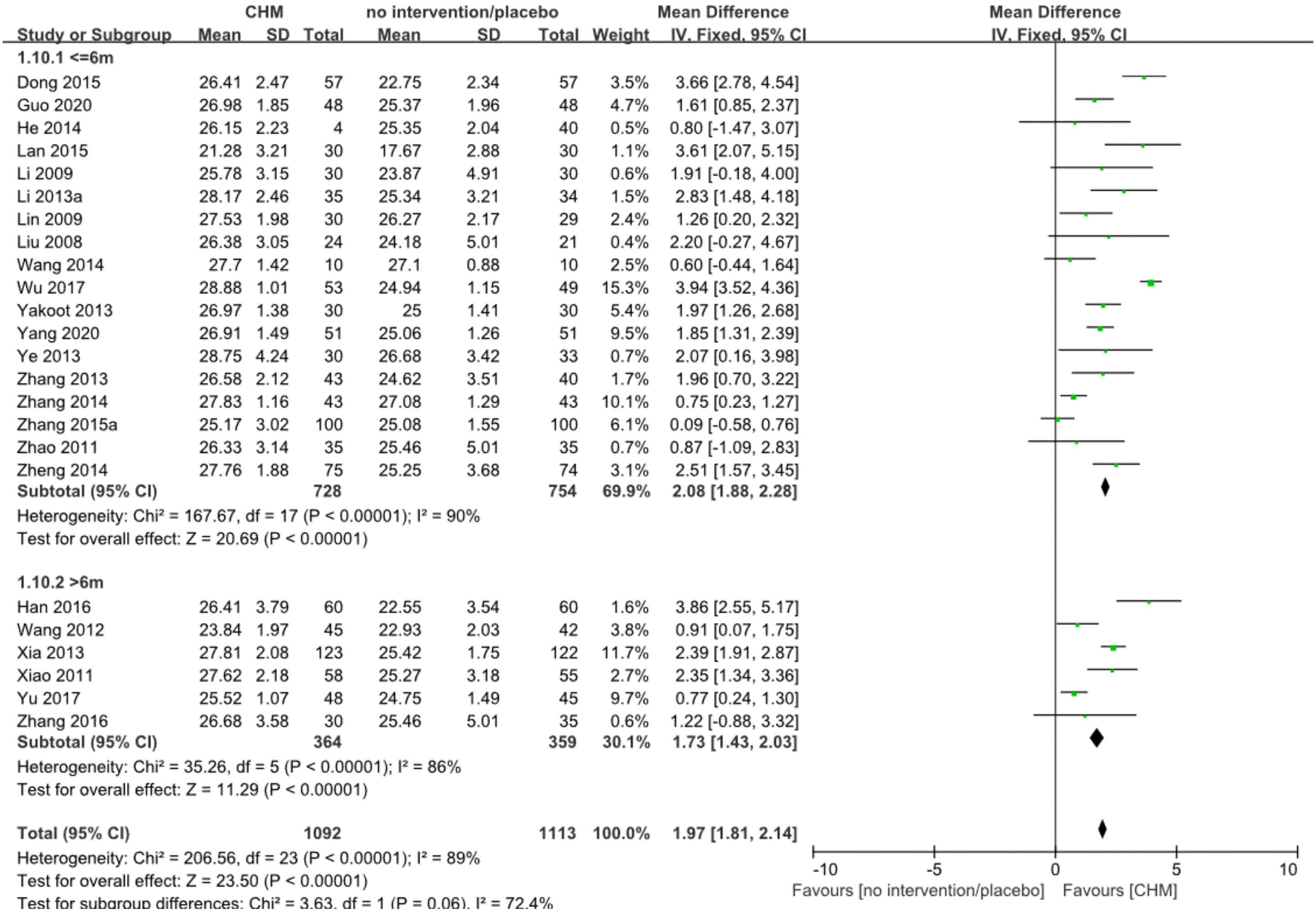

Supplementary Fig. 5. Forest plot of subgroup analyses of comparing trials with different treatment duration of Chinese herbal medicine versus no intervention/ placebo, outcome: MMSE

Study or Subgroup	CHM Mean	CHM SD	CHM Total	no intervention/placebo Mean	no intervention/placebo SD	no intervention/placebo Total	Weight	Mean Difference IV, Fixed, 95% CI
**1.11.1 <=6m**								
Guo 2020	26.84	1.94	48	25.41	2.06	48	17.3%	1.43 [0.63, 2.23]
He 2014	26.95	1.64	40	25.35	2.04	40	16.8%	1.60 [0.79, 2.41]
Su 2015	27.63	6.57	50	20.93	7.89	50	1.4%	6.70 [3.85, 9.55]
Wang 2014	25.8	1.69	140	24.8	1.81	10	8.3%	1.00 [-0.16, 2.16]
Zhang 2010	21.8	2.28	9	16.4	3.75	9	1.3%	5.40 [2.53, 8.27]
Zhang 2013	25.34	2.09	100	20.91	3.34	100	18.6%	4.43 [3.66, 5.20]
Zhang 2015a	24.08	2.53	100	20.91	3.34	100	16.4%	3.17 [2.35, 3.99]
**Subtotal (95% CI)**			**487**			**357**	**80.0%**	**2.63 [2.26, 3.00]**
Heterogeneity: Chi² = 56.44, df = 6 (P < 0.00001); I² = 89%								
Test for overall effect: Z = 13.86 (P < 0.00001)								
**1.11.2 >6m**								
Huang 2015	23.9	3.3	19	21.5	3.9	19	2.1%	2.40 [0.10, 4.70]
Yu 2017	19.39	2.01	48	18.42	1.86	45	17.9%	0.97 [0.18, 1.76]
**Subtotal (95% CI)**			**67**			**64**	**20.0%**	**1.12 [0.38, 1.86]**
Heterogeneity: Chi² = 1.33, df = 1 (P = 0.25); I² = 25%								
Test for overall effect: Z = 2.95 (P = 0.003)								
**Total (95% CI)**			**554**			**421**	**100.0%**	**2.33 [2.00, 2.66]**
Heterogeneity: Chi² = 70.43, df = 8 (P < 0.00001); I² = 89%								
Test for overall effect: Z = 13.72 (P < 0.00001)								
Test for subgroup differences: Chi² = 12.66, df = 1 (P = 0.0004), I² = 92.1%								

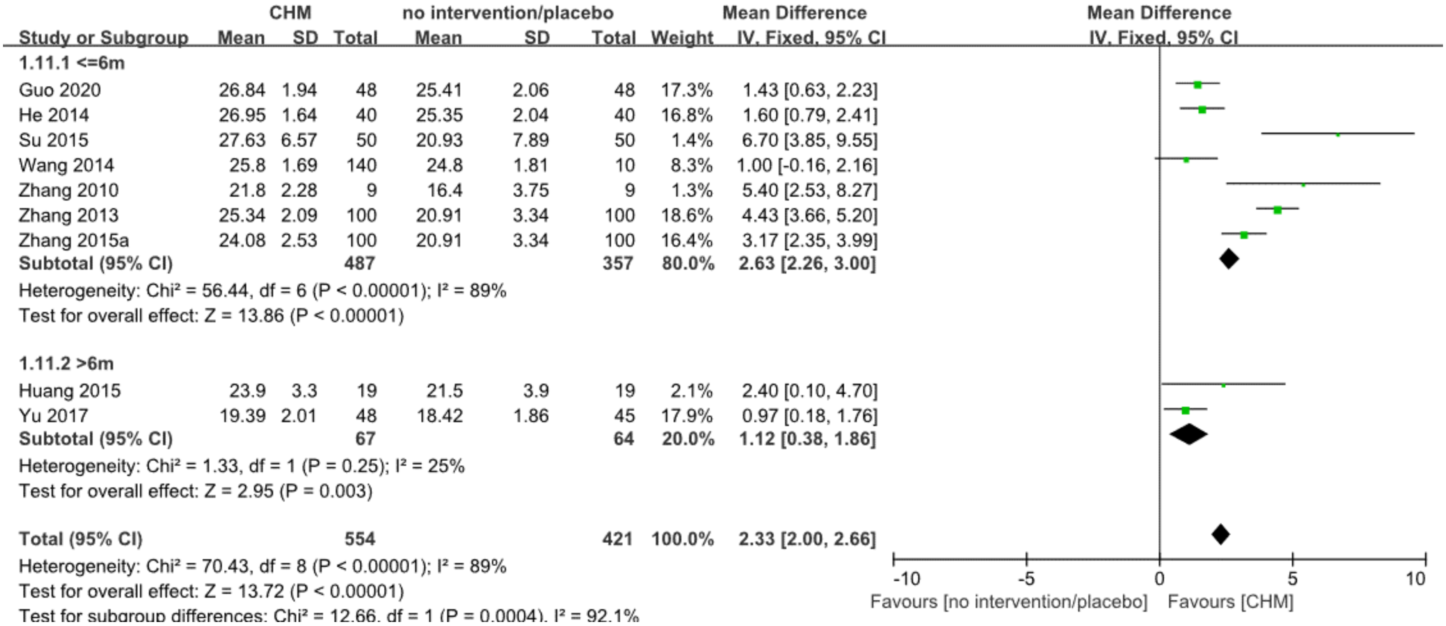

Supplementary Fig. 6. Forest plot of subgroup analyses of comparing trials with different treatment duration of Chinese herbal medicine versus no intervention/ placebo, outcome: MoCA

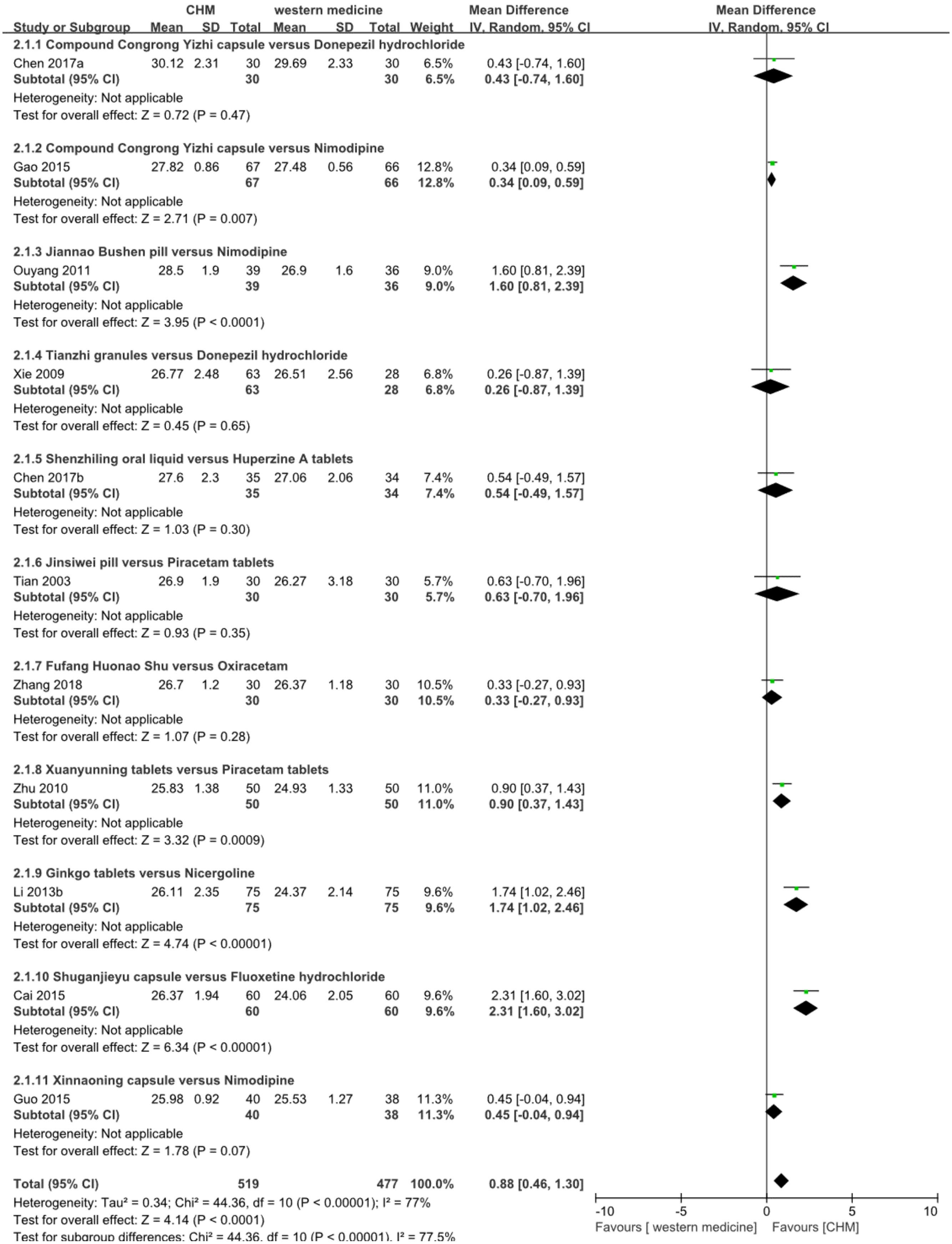

Supplementary Fig. 7. Forest plot of subgroup analyses of different intervention of Chinese herbal medicine versus western medicine, outcome: MMSE

Study or Subgroup	CHM Mean	CHM SD	CHM Total	western medicine Mean	western medicine SD	western medicine Total	Weight	Mean Difference IV, Fixed, 95% CI
**2.4.1 MCI without causes reported**								
Cai 2015	26.37	1.94	60	24.06	2.05	60	5.5%	2.31 [1.60, 3.02]
Chen 2017a	30.12	2.31	30	29.69	2.33	30	2.1%	0.43 [-0.74, 1.60]
Chen 2017b	27.6	2.3	35	27.06	2.06	34	2.7%	0.54 [-0.49, 1.57]
Li 2013b	24.37	2.14	75	26.11	2.35	75	5.5%	-1.74 [-2.46, -1.02]
Tian 2003	26.9	1.9	30	26.27	3.18	30	1.6%	0.63 [-0.70, 1.96]
Xie 2009	26.77	2.48	63	26.51	2.56	28	2.2%	0.26 [-0.87, 1.39]
Zhu 2010	25.83	1.38	50	24.93	1.33	50	10.0%	0.90 [0.37, 1.43]
**Subtotal (95% CI)**			**343**			**307**	**29.6%**	**0.55 [0.24, 0.86]**
Heterogeneity: Chi² = 64.24, df = 6 (P < 0.00001); I² = 91%								
Test for overall effect: Z = 3.48 (P = 0.0005)								
**2.4.2 vascular MCI**								
Gao 2015	27.82	0.86	67	27.48	0.56	66	46.6%	0.34 [0.09, 0.59]
Guo 2015	25.98	0.92	40	25.53	1.27	38	11.6%	0.45 [-0.04, 0.94]
Ouyang 2011	28.5	1.9	39	26.9	1.6	36	4.5%	1.60 [0.81, 2.39]
Zhang 2018	26.7	1.2	30	26.37	1.18	30	7.8%	0.33 [-0.27, 0.93]
**Subtotal (95% CI)**			**176**			**170**	**70.4%**	**0.44 [0.24, 0.64]**
Heterogeneity: Chi² = 8.98, df = 3 (P = 0.03); I² = 67%								
Test for overall effect: Z = 4.28 (P < 0.0001)								
**Total (95% CI)**			**519**			**477**	**100.0%**	**0.47 [0.30, 0.64]**
Heterogeneity: Chi² = 73.57, df = 10 (P < 0.00001); I² = 86%								
Test for overall effect: Z = 5.48 (P < 0.00001)								
Test for subgroup differences: Chi² = 0.35, df = 1 (P = 0.55), I² = 0%								

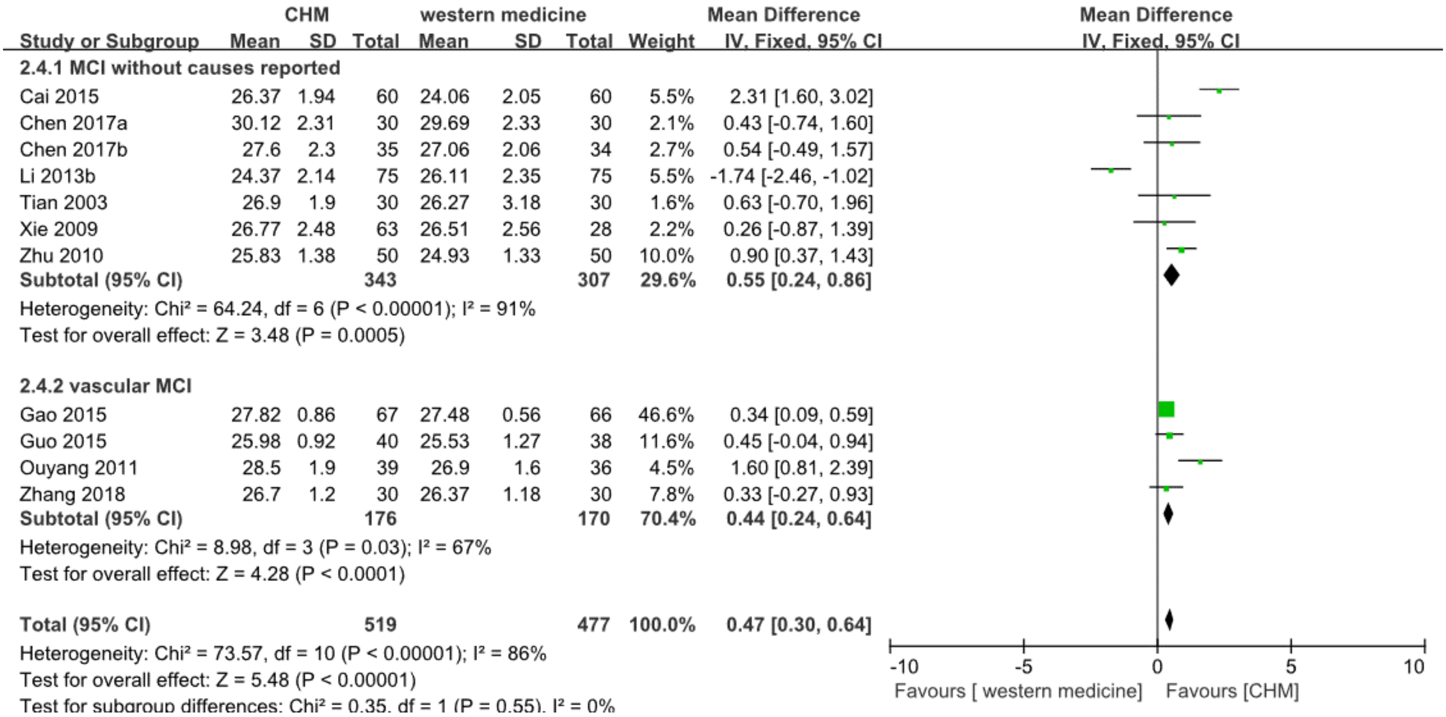

Supplementary Fig. 8. Forest plot of subgroup analyses of different causes of MCI of Chinese herbal medicine versus western medicine, outcome: MMSE

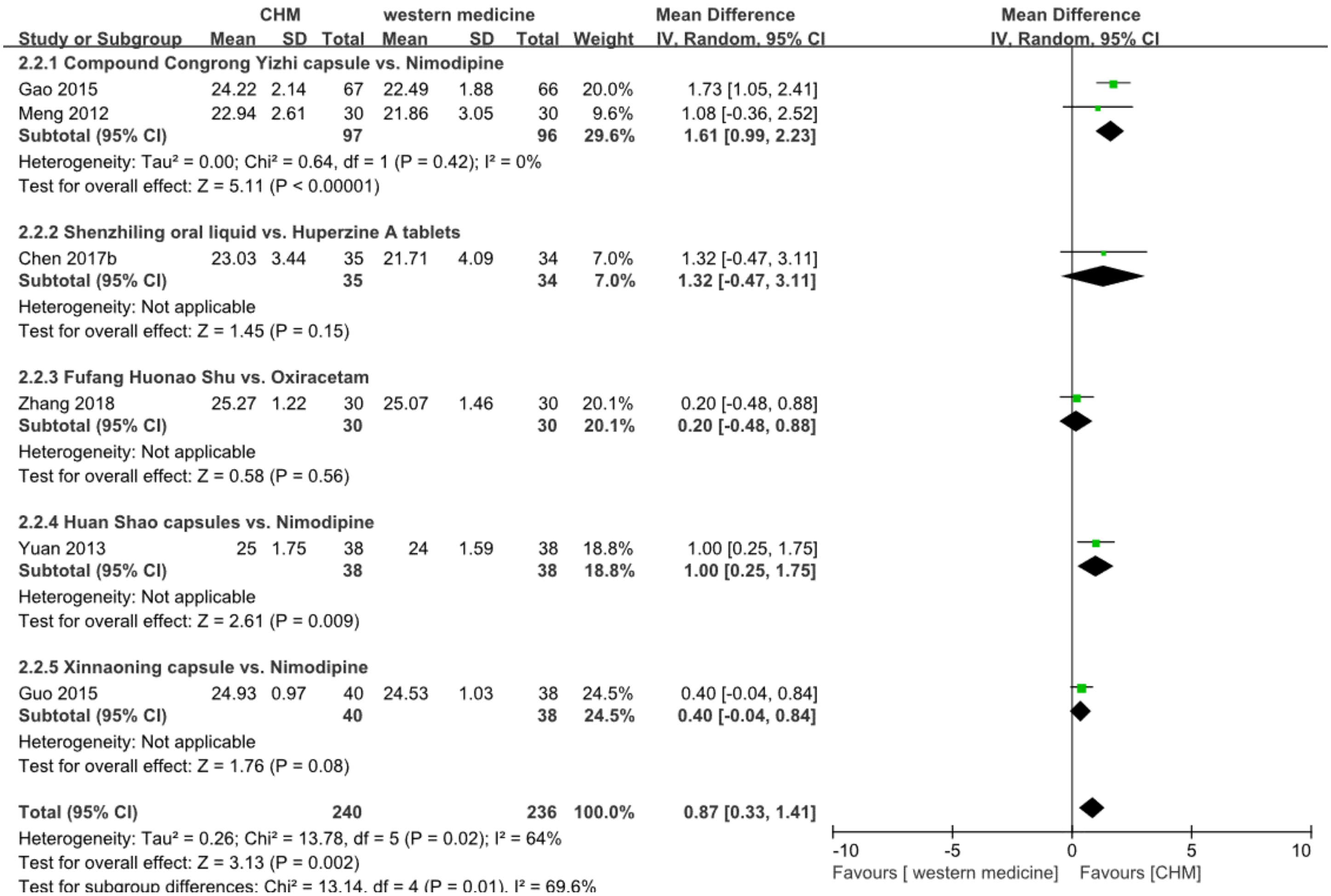

Supplementary Fig. 9. Forest plot of subgroup analyses of different intervention of Chinese herbal medicine versus western medicine, outcome: MoCA

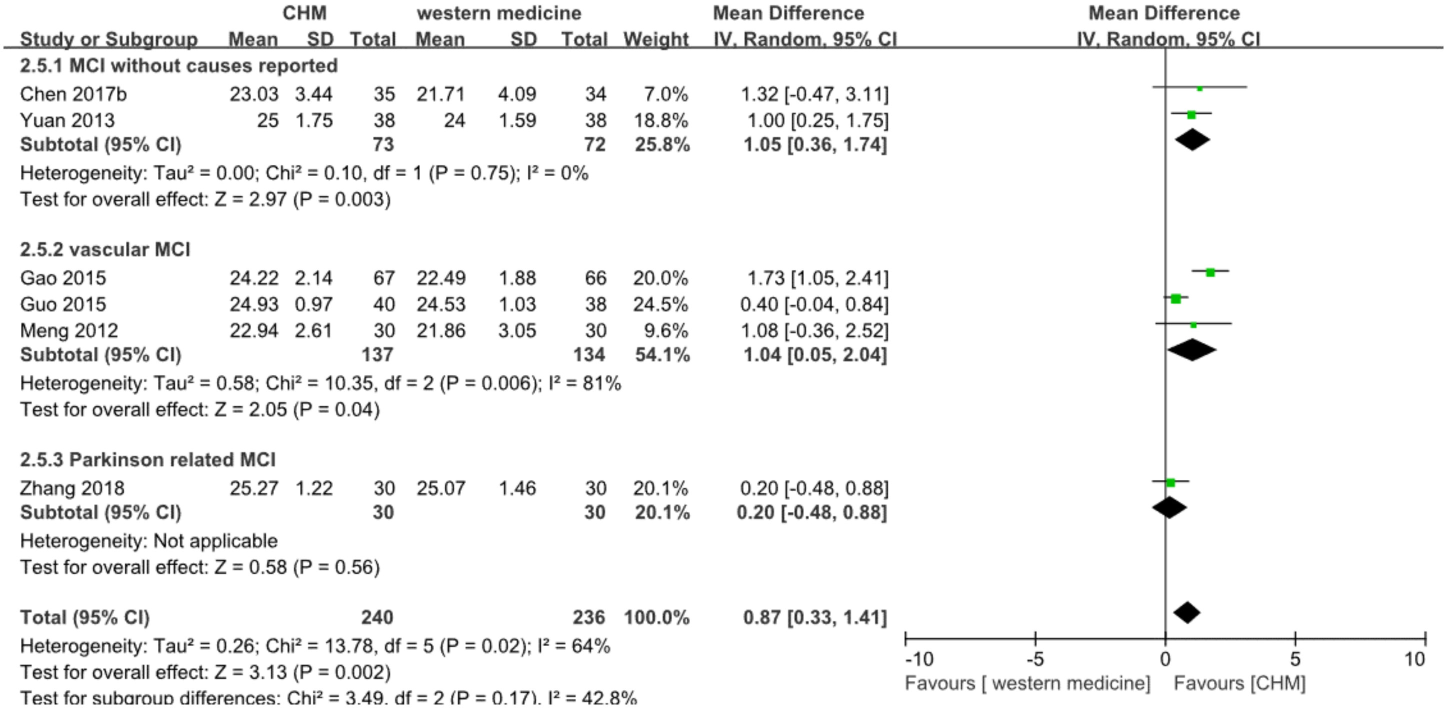

Supplementary Fig. 10. Forest plot of subgroup analyses of different causes of MCI of Chinese herbal medicine versus western medicine, outcome: MoCA

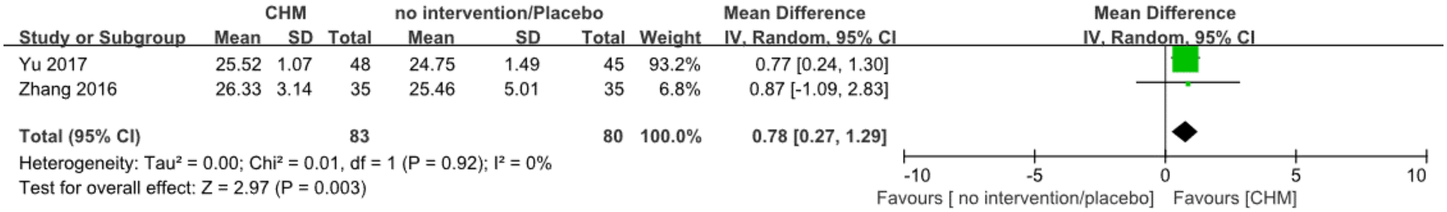

Supplementary Fig. 11. Forest plot of subgroup analyses of amnestic MCI of Chinese herbal medicine versus no intervention/placebo, outcome: MMSE

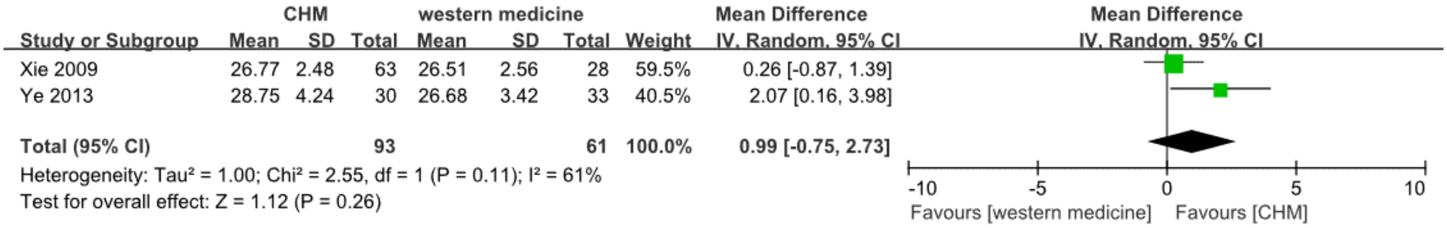

Supplementary Fig. 12. Forest plot of subgroup analyses of amnestic MCI of Chinese herbal medicine versus western medicine, outcome: MMSE

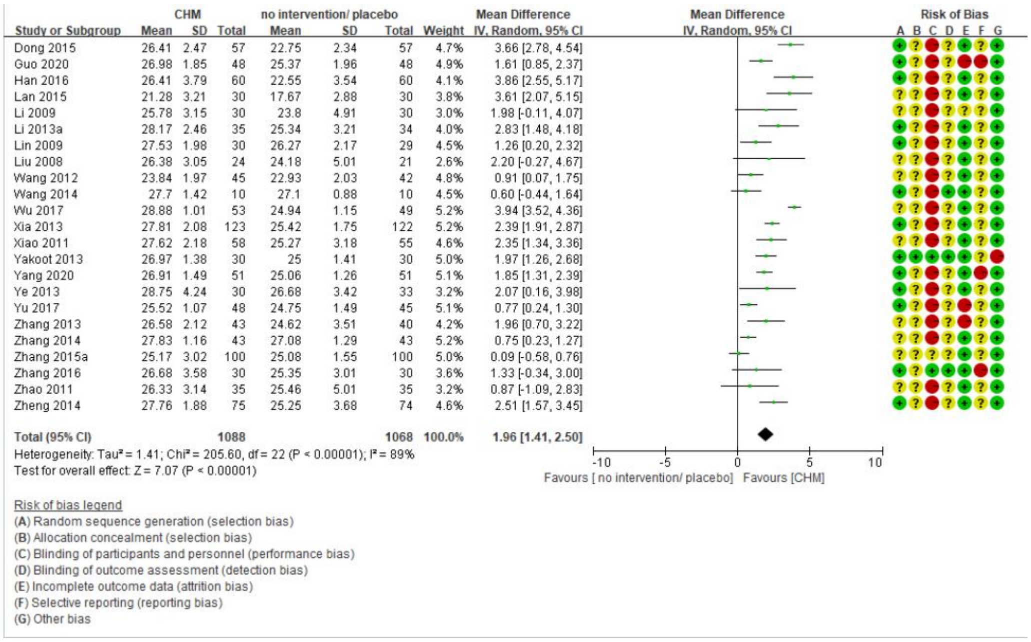

(A)

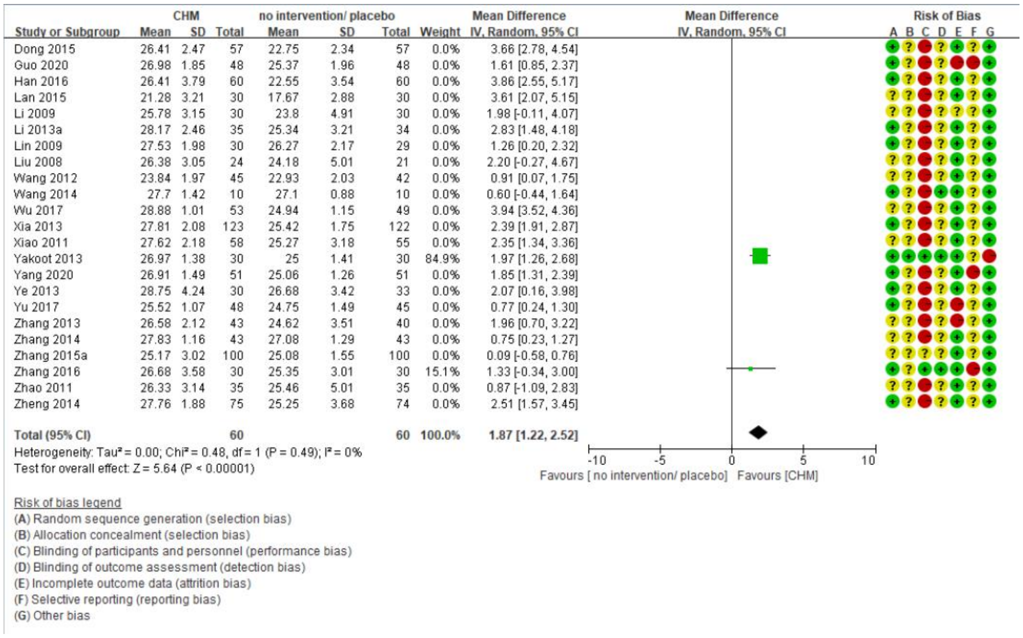

(B)

Supplementary Fig. 13. Forest plots of Chinese patent medicine versus no intervention/ placebo including all trials (A) and including trials with lower risk of bias (B), outcome: MMSE
